# Autophagy selectively clears ER in TNF-α-induced muscle atrophy

**DOI:** 10.1080/27694127.2026.2649064

**Published:** 2026-04-14

**Authors:** Ursula K. Dueren, Alan An Jung Wei, A. Elisabeth Gressler, Simon Rapp, Oliver Popp, Robert Kerridge, Viviana Buonomo, Paolo Grumati, Philipp Mertins, Matthias Selbach, Anna Katharina Simon, Thomas Sommer

**Affiliations:** a Max Delbrück Center for Molecular Medicine in the Helmholtz Association, Berlin, Germany; b Institute for Biology, Humboldt-University zu Berlin, Berlin, Germany; c Proteomics Platform, Max Delbrück Center for Molecular Medicine, Berlin, Germany; d Telethon Institute of Genetics and Medicine, Pozzuoli, Italy; e Department of Clinical Medicine and Surgery, Federico II University, Naples, Italy; f Berlin Institute of Health at Charité - Universitätsmedizin Berlin, Berlin, Germany; g Charité, Universitätsmedizin Berlin, Berlin, Germany; h Institute for Biomedical Translation, Hannover, Germany

**Keywords:** Dynamic SILAC, ER-phagy, inflammation, muscle atrophy, proteostasis, TNF-α, autophagy

## Abstract

Skeletal muscle atrophy is a pathological condition characterized by the progressive loss of muscle mass and function, driven by factors such as disuse, inflammation, and aging. While the ubiquitin-proteasome system is established as the central mediator of myofibrillar protein degradation, the role of selective autophagy and the degradation of organelles remains underexplored in this context. To address this, we employed a quantitative, time-resolved *in vitro* analysis of protein synthesis and degradation in C2C12 myotubes undergoing TNF-α-induced atrophy, using dynamic Stable Isotope Labeling by Amino Acids in Cell Culture (SILAC) coupled with LC-MS/MS. Our data challenges the classical view of atrophy as a uniform, degradation-centric process. Instead, we reveal temporally distinct patterns of selective protein turnover, including differential degradation of myofibrillar, ribosomal, and endoplasmic reticulum (ER)-resident proteins. Early atrophy is characterized by suppressed short-term protein synthesis, increased ubiquitin-ligase expression, proteasomal activation, and ribosome turnover. In contrast, late atrophy features proteasome-dependent myofibrillar protein degradation, selective synthesis, and degradation of mitochondrial and cytoplasmic ribosomes, indicative of metabolic adaptation. Moreover, we identify a temporal shift in autophagic selectivity: from ER homeostasis to a stress-induced ER-degradation program. Notably, autophagy inhibition during atrophy leads to the accumulation of ER-phagy receptors Tex264 and Calcoco1, implicating ER-phagy as a key contributor to atrophic remodeling and highlighting receptor-mediated selective autophagy as a regulatory axis in muscle proteostasis. By elucidating the role of ER-phagy, this study opens avenues for therapeutic interventions targeting proteostasis in inflammation-induced muscle-wasting, contributing to a refined understanding of muscle atrophy beyond proteasomal degradation, particularly in acute inflammatory conditions such as sepsis.

## Introduction

Skeletal muscle atrophy is a pathological condition characterized by the loss of muscle mass and strength, driven by diverse stimuli including disuse, malnutrition, acute and chronic inflammation, and aging. This process has profound implications for overall health, leading to impaired mobility, metabolic dysregulation, and increased disease susceptibility. Muscle atrophy has been attributed to an imbalance in proteostasis, where protein degradation surpasses synthesis, primarily affecting myofibrillar proteins and cellular organelles [1,2]. This long-standing paradigm is supported by several observations. First, the majority of muscle proteins are myofibrillar, and they undergo significant breakdown during atrophying conditions alongside an activation of proteolytic systems [3,4]. Additionally, suppressed protein synthesis via the Akt/mTOR pathway and activation of Forkhead-Box-Protein (FoxO) signaling further contribute to muscle loss by upregulation of E3 ubiquitin ligases such as Muscle RING-finger protein-1 (MuRF1) and Atrogin-1, promoting ubiquitin proteasome system (UPS)-dependent degradation of sarcomeric proteins [2,5]. Despite that, denervation model studies in rats have shown a more nuanced process with both increased protein degradation and elevated protein synthesis rates [6]. This complexity underscores the need for a time-resolved approach to observe protein dynamics across different atrophy-inducing conditions, fiber-type compositions and model organisms, rather than assuming a uniform degradation-centric model.

C2C12 mouse skeletal muscle myotubes are widely used as a skeletal muscle model, allowing for a controlled investigation of atrophy-inducing stimuli such as pro-inflammatory cytokines like tumor necrosis factor-alpha (TNF-α). This leads to the activation of multiple pathways including FoxO, nuclear factor kappa B (NF-κB), and transcription factor EB (TFEB) signaling, encompassing protein degradation and autophagy [7–12]. TNF-α-driven inflammation is a key mediator of acute muscle wasting in conditions such as sepsis, where it exacerbates proteolytic activity and disrupts proteostasis. It also plays a role in chronic muscle-wasting diseases like cachexia [13,14] and age-related sarcopenia [15–17]. Sepsis-associated muscle wasting (SAMW) affects up to 40% of critically ill, ICU-admitted patients [18] can manifest as early as 48 h after onset [19]. Unlike disuse atrophy, which is often reversible, SAMW is associated with long-term functional deficits and involves sustained activation of proteolytic pathways [20]. Despite its clinical relevance, there are no effective pharmacological treatments.

Most studies focus on the influence of the UPS on atrophy progression, whereas other proteolytic systems like the autophagy/lysosome pathway (primarily referring to macroautophagy, distinct from chaperone-mediated autophagy (CMA) and microautophagy) have emerged as a critical but still underexplored regulator of muscle proteostasis. Hereafter, for simplicity, we refer to macroautophagy as “autophagy.” Autophagy plays a central role in the selective degradation of damaged proteins and organelles through the formation of double-membrane vesicles (autophagosomes) that engulf and thereby deliver substrates to lysosomes for degradation. While basal autophagy is essential for cellular homeostasis, autophagy is upregulated upon a variety of atrophying stimuli like acute inflammation-induced atrophy [7,12] and denervation [21]. Suppression of autophagy – shown by a muscle-specific deletion of a key autophagy gene, *Atg7* – results in muscle atrophy, accompanied by disorganization of the sarcomere, abnormal mitochondria, sarcoplasmic reticulum distension and formation of aberrant membranous structures [22]. These morphological features suggest that the loss of selective organelle clearance pathways such as mitophagy and ER-phagy may underlie functional degradation in muscle. In sepsis-induced muscle atrophy, autophagy is robustly activated [23], reinforcing its early and context-dependent role in regulating muscle proteostasis. On the other hand, several studies suggest that activation of autophagy during catabolic conditions can aggravate muscle loss. Loss-of-function mutations in Jumpy, which is a phosphatase that antagonizes the autophagy-inducing kinase VPS34, thereby limiting autophagosome formation, is associated with centronuclear myopathy [24].

While proteasomal degradation is based on well-defined ubiquitin targeting in muscle atrophy, selective autophagy, particularly its receptor-guided cargo recognition mechanisms for organelles like the ER or mitochondria, remains poorly resolved. Given the dual role of autophagy in both preserving and degrading muscle components, a deeper understanding of its temporal dynamics and substrate specificity during atrophy is crucial.

This study provides a time-resolved, quantitative analysis of protein synthesis and degradation dynamics during TNF-α-induced atrophy in C2C12 myotubes. Using dynamic Stable Isotope Labeling with Amino Acids in Cell Culture (dynamic SILAC) [25] coupled with LC-MS/MS, we analyzed the proteome landscape of early and late TNF-α-induced atrophic response while also differentiating between degraded and newly synthesized proteins. This manuscript builds upon our previously published preprint [26]. Contrary to the conventional view that atrophy is primarily driven by reduced protein synthesis and increased protein degradation, our findings reveal a more nuanced remodeling of the proteome, characterized by selective regulation of specific protein groups. Notably, while the biosynthesis of ribosomal proteins is suppressed during atrophy, mitochondrial ribosomal protein synthesis is upregulated. We further explore the role of proteolytic systems, particularly autophagy, in maintaining proteostasis under atrophic conditions. Our results indicate that autophagy selectively degrades specific subsets of proteins: while myofibrillar proteins are spared, ER-resident proteins are *bona fide* autophagic cargos. We thereby uncover the potential role of ER-phagy in atrophic muscle remodeling. Understanding the contribution of the understudied autophagy pathway to skeletal muscle atrophy may uncover novel therapeutic targets for muscle-wasting disorders and bridge the knowledge gap between protein turnover regulation and metabolic adaptations in muscle atrophy.

## Results

### Dynamic SILAC identifies protein turnover changes in TNF-α-induced muscle atrophy

**Figure 1.**
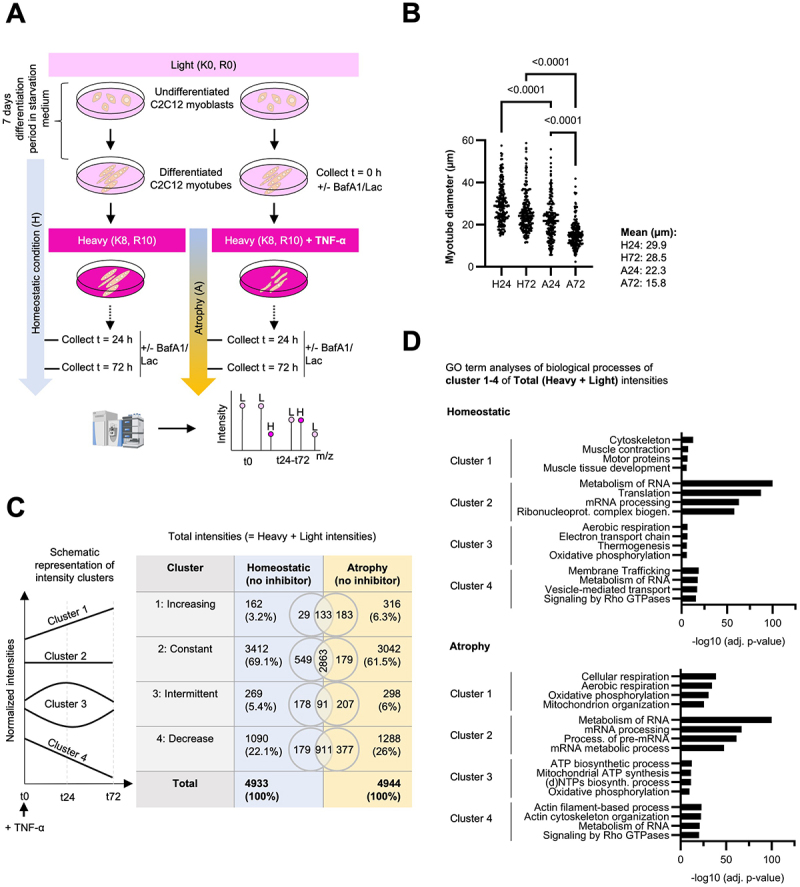
Dynamic SILAC reveals distinct sets of proteins affected in their turnover upon TNF-α-induced muscle atrophy in C2C12 cells. (A) Experimental workflow: C2C12 myoblasts were differentiated for seven days in light medium (K0 = Lysine 0 [^12^C_6_, ^14^N_2_]; R0 = Arginine 0 [^12^C_6_, ^14^N_4_]) into myotubes (= t0). Medium was then replaced with heavy medium (K8 = Lysine 8 [^13^C_6_, ^15^N_2_]; R10 = Arginine 10 [^13^C_6_, ^15^N_4_]) for 24 or 72 h (t24, t72), with or without TNF-α to induce atrophy. Proteolytic inhibitors (BafA1: Bafilomycin A1 inhibiting autophagy; Lac: Lactacystin inhibiting the UPS) were administered for 3 h prior to cell harvest, and cells were analyzed using LC-MS/MS. *n* = 3 replicates. (B) Myotube diameter was measured in differentiated C2C12 myotubes under homeostatic conditions at 24 h (H24; day 8 of differentiation) and 72 h (H72; day 10), as well as following TNF-α-induced atrophy at corresponding time points (A24 and A72). Measurements were performed using ImageJ on 209 individual myotubes per condition, quantified from 15-20 randomly selected images. Statistical significance was determined using ordinary one-way ANOVA with multiple comparisons (GraphPad Prism). Images used were acquired from OPP-stained samples from Figure 2. (C) Protein clustering based on total intensity dynamics of non-inhibitor treated homeostatic and atrophying cells: Proteins were categorized into four clusters according to their normalized total intensity values (light + heavy) at t0, t24, and t72. Percentages of protein counts were rounded up. (D) Proteins of each cluster of total intensities were subjected to GO term enrichment (biological processes) analysis.

To investigate the details of proteostasis in skeletal muscle during homeostatic and atrophying conditions, we employed a dynamic Stable Isotope Labeling with Amino Acids in Cell Culture (dynamic SILAC) approach [25] in C2C12 mouse skeletal muscle cells, both in homeostatic conditions and TNF-α-induced atrophy (Figure 1(A)). Our study focuses on *de novo* protein synthesis and degradation dynamics by assessing the contributions of the autophagy-lysosome pathway (referred to as autophagy from now on) and the UPS to these processes. To delineate their roles in protein turnover, we utilized pharmacological inhibitors prior to cell harvest – Bafilomycin A1 (BafA1) for inhibiting autophagosome-lysosome fusion and thus autophagy and Lactacystin (Lac) for inhibiting the UPS. The experimental workflow began with the differentiation of C2C12 myoblasts into mature myotubes in a light-labeled medium (containing K0 = Lysine 0 [^12^C_6_, ^14^N_2_]; R0 = Arginine 0 [^12^C_6_, ^14^N_4_]). After 7 days of differentiation, myofibrillar structures are fully developed (baseline t0). At this point, the cells were switched to a heavy-labeled medium (containing K8 = Lysine 8 [^13^C_6_, ^15^N_2_]; R10 = Arginine 10 [^13^C_6_, ^15^N_4_]), ensuring that every newly synthesized protein would incorporate heavy amino acids and be detected as “heavy-labeled” in mass spectrometry. This enabled us to track protein synthesis by following the accumulation of heavy-labeled peptides, while protein degradation was inferred from the disappearance of preexisting light-labeled proteins over time. The cells were harvested after 24 h (t24) and 72 h (t72) to evaluate protein turnover in differentiated myotubes over a three-day period. To assess the atrophic influence on proteostasis, we followed the same labeling approach but added TNF-α at the time of the medium switch (day 7) to the culture to induce muscle atrophy. We confirmed that myotube diameter decreases significantly at both t24 and t72 following TNF-α treatment, validating the onset and progression of the atrophic phenotype (Figure 1(B)). Cells were again harvested 24 h and 72 h post-pulse. The collected cells were analyzed using liquid chromatography-tandem mass spectrometry (LC-MS/MS). We refer to 24 h and 72 h of TNF-α treatment as “early” and “late” time points, respectively, within the confines of this acute inflammatory model. These labels are operational, reflecting the controlled design of our *in vitro* system rather than a complete phenotypic timeline of muscle atrophy. While this model does not recapitulate the long-term degeneration seen in chronic atrophy, this early window is highly relevant to acute muscle-wasting contexts such as sepsis, where muscle loss is already detectable early at 48 h [27] and 96 h [28].

Principal component analyses (PCA) reveal distinct clustering patterns between homeostatic and atrophying conditions in the heavy fraction (newly synthesized proteins) (Figure S1A-C). In contrast, in the light fractions (degraded proteins), samples treated with TNF-α for 24 h (A24) cluster rather closely with homeostatic controls at the same time point, with separation becoming more apparent after 72 h of atrophy (A72) (Figure S1A). Inhibition of the UPS with Lac significantly impacts the overall proteome in both heavy and light fractions, as indicated by distinct clustering across time points (Figure S1B,C). Additionally, also in both heavy and light fractions, samples treated with BafA1 and those without proteolytic inhibitor treatment cluster closely together (Figure S1C). In total, nearly 5000 proteins were detected. Normalized light and heavy intensities were summed up to calculate the total intensities of individual proteins at time points t0, t24, and t72. Based on the temporal profiles of their total intensities, we sorted the proteins into four distinct clusters (Figure 1(C)): (1) those with steadily increasing intensities from t0 to t72; (2) those with constant intensities, defined as exhibiting no more than a 10% variation relative to the maximum change intensity; (3) those with intermittent intensities, characterized by an initial drop or rise at t24 followed by a return to baseline levels at t72; and (4) those with steadily decreasing intensities from t0 to t72. Interestingly, our analyses did not reveal major differences in the overall percentages of proteins with increasing or decreasing intensities between homeostatic and atrophying conditions.

However, Gene Ontology (GO) enrichment analysis (Figure 1(D)) of each cluster revealed distinct functional associations in atrophying and homeostatic cells, particularly in clusters 1 and 4. In homeostatic conditions, as expected, proteins that increased their intensity over time (cluster 1) were primarily associated with the cytoskeleton, tissue development, and muscle contraction, whereas in atrophy, unexpectedly, they were linked to mitochondrial energy metabolism. Proteins summed up in cluster 4 (decreasing) in homeostatic conditions were associated with membrane trafficking and vesicle-mediated transport, while in atrophy, they were predominantly related to actin filament-based process and actin cytoskeleton organization. Clusters 2 and 3 comprise similar GO terms for both, homeostatic conditions and in atrophy. While cluster 2 (constant expression) was associated with RNA metabolism and translation, cluster 3 (intermittent expression) GO terms related to oxidative phosphorylation (OXPHOS) and mitochondrial ATP synthesis. We decided to further investigate these pathways by measuring translation rates under both conditions.

### Short-term global translation inhibition in TNF-α-induced atrophy

**Figure 2.**
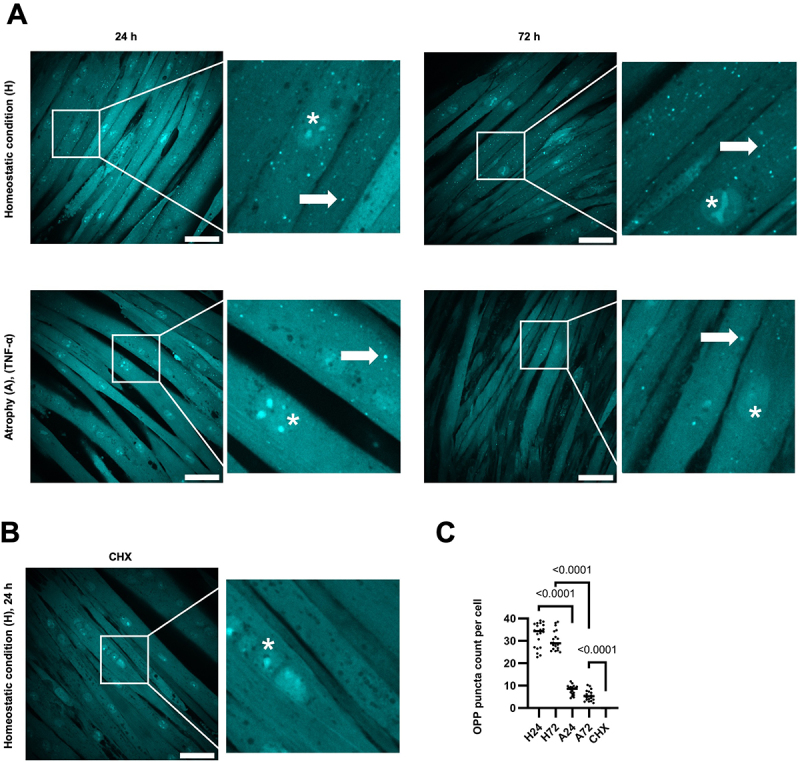
TNF-α-induced atrophy leads to acute translation inhibition. (A) OPP (O-propargyl-puromcyin) labeling of differentiated myotubes (homeostatic at 24 h (H24) and 72 h (H27)) and TNF-α-induced atrophying C2C12 cells (24 h (A24) and 72 h (A72)) was used to measure protein synthesis rates at time points t24 and t72. Arrows indicate puncta representing newly translated proteins. Asterisks indicate nuclei in differentiated, multinucleated myotubes. (B) Cycloheximide (CHX) was employed for 90 min as a control to inhibit protein synthesis. Puncta were manually counted across 20 images per condition, each containing over 200 cells. (C) Statistical significance was assessed using one-way ANOVA followed by Tukey’s multiple comparisons test. Scale bar: 60 µm.

To determine short-term global translation rates, we utilized the Click-iT® Plus OPP (O-propargyl-puromycin) Protein Synthesis Assay to visualize protein synthesis in both homeostatic and TNF-α-induced atrophying C2C12 myotubes (Figure 2(A)). During OPP labeling (30 min), newly synthesized proteins get fluorescently tagged with a puromycin analog and appear as puncta in the cell, which serve as quantification base for protein synthesis rates. Background cytosolic staining might appear due to incomplete washing, nonspecific binding or excess fluorophore. As expected, treatment with the protein synthesis inhibitor cycloheximide (CHX) effectively abolished protein synthesis (Figure 2(B)), validating the specificity of the OPP assay. In homeostastic conditions (H24, H72), protein synthesis remains high over the time course. In contrast, we observed significantly lower acute protein synthesis rates in atrophying myotubes compared to homeostatic myotubes, with no significant difference (Figure 2(C)) in protein synthesis rates between early (A24, 24 h) and late (A72, 72 h) atrophy.

### Dynamic SILAC analysis identifies early- and late-phase protein degradation and synthesis signatures during TNF-α-induced atrophy

**Figure 3.**
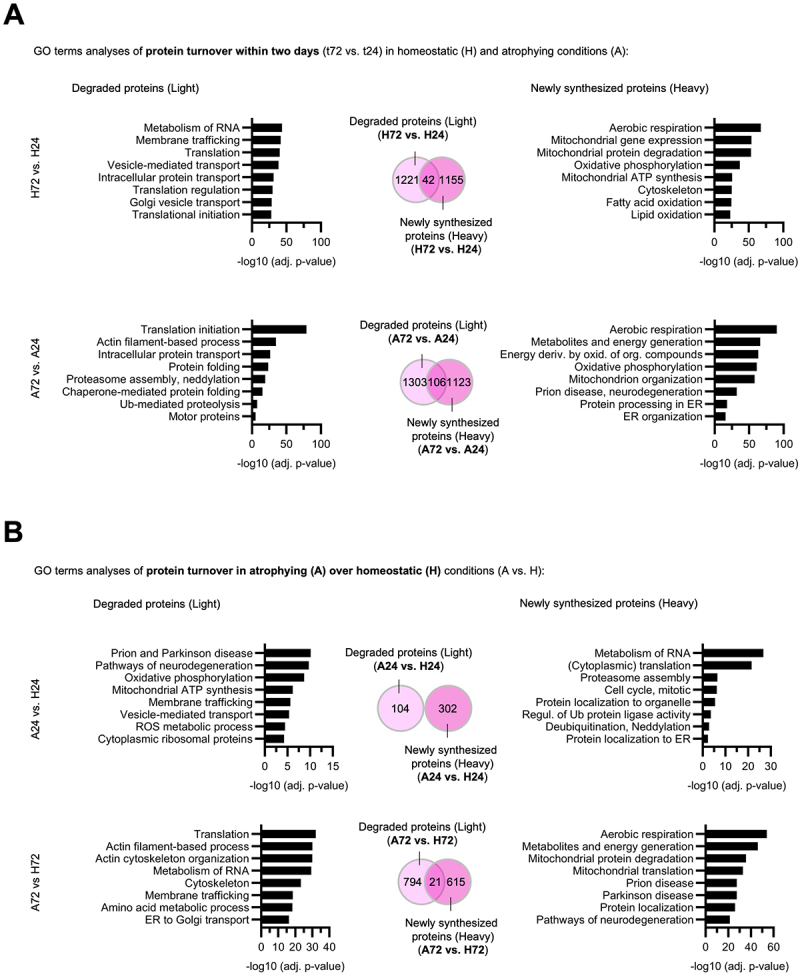
Dynamic SILAC analysis identifies early and late-phase protein degradation and synthesis signatures during TNF-α-induced atrophy. (A-B) Stepwise ANOVA comparisons were performed on heavy and light dynamic SILAC samples to assess significant protein degradation and synthesis rates across and between homeostatic (H) and atrophic (A) conditions. Proteins with log_2_FC < 0 and adj. *p*-value < 0.05 in the light channel were classified as significantly degraded, while proteins with log_2_FC > 0 and adj. *p*-value < 0.05 in the heavy channel were classified as significantly newly synthesized. Numbers depict total amounts of significantly regulated proteins in respective dataset comparison. GO term analyses (biological processes) of (A) protein turnover within two days (t72 vs. t24) in homeostatic and atrophying samples and (B) protein turnover in atrophying over homeostatic samples (A vs. H). Numbers in Venn diagrams depict amount of significantly regulated proteins in respective dataset comparison. Light pink resembles light channel, dark pink resembles heavy channel.

Proteome analysis via dynamic SILAC enables the distinction between newly synthesized proteins (heavy channel) and preexisting proteins (light channel), eliminating reliance on total intensity measurements. To assess protein turnover over time in both atrophic and homeostatic conditions, we performed a differential abundance analysis using limma [29] with moderated t-tests and ANOVA (empirical Bayes), applying a *p* ≤ 0.05 threshold and Benjamini–Hochberg correction. We compared the abundance of preexisting proteins (light channel) at 72 h (t72) relative to 24 h (t24). Proteins showing a significant reduction (log_2_FC < 0, adj. *p*-value < 0.05) were classified as actively degraded. Similarly, newly synthesized proteins were identified by comparing the heavy channel at t72 to t24, with significant accumulation (log_2_FC > 0, adj. *p*-value < 0.05), indicating active synthesis (Figure 3(A)). Interestingly, we observed no major changes in the overall number of newly synthesized or degraded proteins during 2 days of atrophy (A72 vs. A24) compared to 2 days of homeostatic conditions (H72 vs. H24). Therefore, we decided to perform GO analyses to determine differentially regulated proteins: During homeostatic conditions (H72 vs. H24), we identified 1197 newly synthesized proteins, primarily enriched in GO terms related to mitochondria-dependent energy metabolism but also cytoskeleton, and 1263 degraded proteins, associated with RNA metabolism, membrane trafficking, and protein transport. In contrast to that, within 2 days of atrophy (A72 vs. A24), 1229 proteins were newly synthesized and 1409 were degraded. GO analysis revealed that proteins significantly synthesized during 2 days of atrophy were also involved in mitochondrial energy production, predominantly OXPHOS), but interestingly, also proteins involved in processing in the ER, and ER organization were increasingly synthesized. In contrast to homeostatic conditions, degraded proteins during atrophy were associated with translation, protein transport and folding, actin filament-based processes, cytoskeletal organization, proteasome assembly, ubiquitin-mediated proteolysis, and motor proteins. To sum up, despite similar amounts of overall proteins affected by protein turnover, we could clearly observe a distinctly regulated proteome landscape with atrophy-specific synthesis of proteins enriched in ER organization and protein processing. Degraded proteins during atrophy were related to translation, cytoskeletal structure, protein folding and proteasome-related pathways, indicating a reprogramming of cellular organization and maintenance mechanisms under prolonged atrophic stimulus.

To distinguish early and late atrophic responses relative to homeostatic conditions, we also compared A24 vs. H24 and A72 vs. H72 (Figure 3(B)). Again, proteins showing a significant reduction in the light channel (log_2_FC < 0, adj. *p*-value < 0.05) were classified as actively degraded, whereas newly synthesized proteins were identified with log_2_FC > 0 and an adj. *p*-value of <0.05. In early TNF-α response (A24 vs. H24), 302 newly synthesized proteins specific to atrophy were detected, with no overlap with the 104 degraded proteins identified. GO analysis of degraded proteins in A24 vs. H24 highlighted pathways associated with protein misfolding and aggregation (neurodegeneration-related GOs), ATP synthesis, protein transport, ROS metabolic processes, and cytoplasmic ribosomal protein degradation. Newly synthesized proteins in A24 vs. H24 were linked to cytoplasmic translation, cell cycle regulation, UPS components, and protein localization to the ER. In late atrophy (A72 vs. H72), the number of newly synthesized proteins specific to atrophy nearly doubled (626) and degraded proteins increased to 815. GO analyses of degraded proteins in late TNF-α response depicted pathways related to translation, actin cytoskeleton, membrane trafficking and amino acid metabolism, whereas newly synthesized proteins were related to cellular energy metabolism, degradation and translation of mitochondria, neurodegeneration-related GOs and protein localization. To sum up, comparison of the early and late TNF-α-induced atrophic proteome to the proteome of homeostatic cells revealed a marked increase in both newly synthesized and actively degraded proteins, indicating a shift in the cellular program from 24 h to 72 h of TNF-α exposure toward enhanced protein turnover, mitochondrial remodeling and stress adaptation.

### Selective degradation and impaired synthesis of myofibrillar proteins during TNF-α-induced muscle atrophy

**Figure 4.**
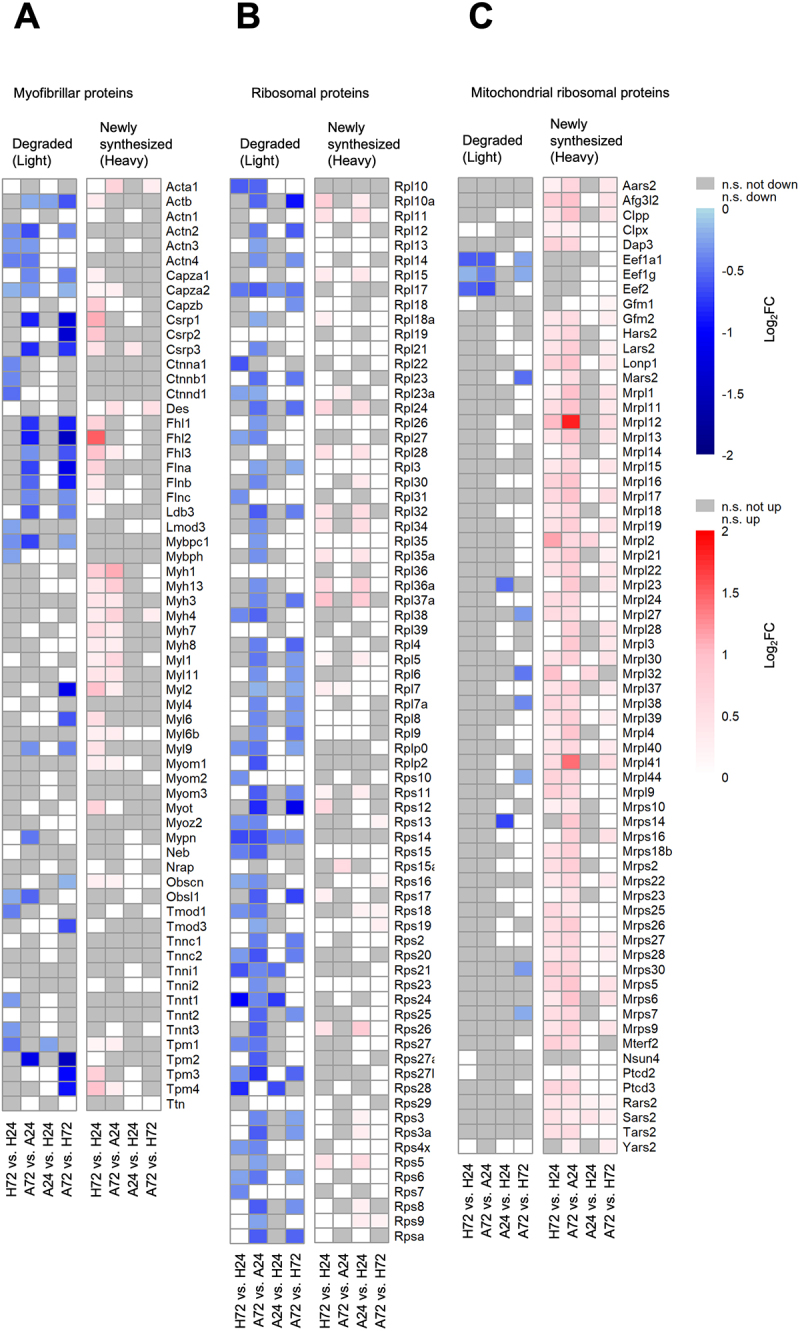
Differential regulation of myofibrillar components, ribosomal and mitochondrial ribosomal proteins upon TNF-α -induced atrophy. (A-D) Stepwise ANOVA comparisons were performed on heavy and light dynamic SILAC samples to assess significant protein degradation and synthesis rates across and between homeostatic (H) and atrophic (A) conditions. Proteins with log_2_FC < 0 and adj. *p*-value < 0.05 in the light channel were classified as significantly degraded, while proteins with log_2_FC > 0 and adj. *p*-value < 0.05 in the heavy channel were classified as significantly newly synthesized. Heatmap of degraded and newly synthesized (A) myofibrillar (B) ribosomal and (C) mitochondrial ribosomal proteins. White: not significantly up/downregulated; grey: not significant.

Skeletal muscle atrophy is characterized by the breakdown of myofibrillar components, which we could also observe in our previous GO analyses (Figure 3(B), “actin-filament-based processes,” “motor proteins”). However, an in-depth analysis focusing on the specific proteins targeted for degradation during different atrophy stimuli remains lacking. To address this gap, we categorized myofibrillar proteins into the previously defined clusters (Figure 1(C)) of total intensities to assess their absolute dynamics under homeostatic and atrophic conditions (Figure S2A). Generally, we could observe that the majority of myofibrillar components show stable intensities from t0 to t72 in both conditions (therefore assigned to cluster 2). In both conditions, the majority of myofibrillar components were assigned to cluster 2, indicating stable intensities from t0 to t72. However, in atrophy, as expected, a greater proportion of myofibrillar proteins were sorted into cluster 4 (decreasing intensities from t0 to t72), reflecting overall decrease. Conversely, fewer myofibrillar proteins were assigned to cluster 1 (increasing intensities from t0 to t72) in atrophy. Notably, Myh4, one of the myosin heavy-chain proteins, which acts as a critical component to sustain contraction, was the only myofibrillar protein that was assigned to cluster 1 under atrophic conditions but was classified in cluster 2 (constant intensities) under homeostatic conditions. These findings prompted us to further examine degradation (Figure 4(A) left) and synthesis (Figure 4(A) right) of individual myofibrillar components in TNF-α-induced atrophying conditions and how these changes compare to homeostatic conditions instead of observing total intensities (Figure S2A). Proteins in close proximity to actin filaments (Actn1-4), including Capza1,2 and Flna-c but also proteins located at the M band (Fhl1-3, Myl, Tpm2-4) exhibited pronounced degradation in atrophic conditions (A72 vs. H72), whereas myofibrillar protein degradation was not abolished in homeostatic conditions (H72 vs. H24) due to normal protein turnover. Myofibrillar protein synthesis in comparisons of atrophy vs. homeostatic condition (A24 vs. H24 and A72 vs. H72) showed no significant upregulation, whereas, in stark contrast, homeostatic conditions (H72 vs. H24) demonstrated high synthesis rates of myofibrillar proteins. However, myofibrillar protein synthesis was not entirely abolished in atrophy. The comparison between late and early atrophy (A72 vs. A24), reflecting synthesis over 2 days of atrophy, revealed some synthesis that was downregulated compared to 2 days of homeostatic conditions, with mostly myosin heavy chain (Myh) proteins again being newly synthesized in late-stage atrophy. Interestingly, all monitored myofibrillar proteins were either newly synthesized or degraded, but not both, suggesting inverse regulation of protein turnover at the single-protein level. In summary, an in-depth analysis of differentially degraded and newly synthesized myofibrillar proteins shows increased degradation in late-stage atrophy and moderate but lower degradation in homeostatic conditions compared to atrophying conditions. In contrast, myofibrillar protein synthesis is reduced in atrophy compared to homeostatic conditions.

### Reduced synthesis of cytoplasmic ribosomal proteins contrasts with increased mitochondrial ribosomal protein synthesis during muscle atrophy

Ribosomal proteins underwent pronounced degradation during 2 days of atrophy (A72 vs. A24), exceeding degradation observed during the equivalent homeostatic period (H72 vs. H24) (Figure 4(B) left), reflecting reduced translation rates (Figure 2). Moreover, degradation rates of ribosomal proteins were notably higher in late atrophy compared to homeostatic conditions (A72 vs. H72) than in early atrophy (A24 vs. H24). Interestingly, ribosomal protein synthesis occurred substantially during the two-day homeostatic period (H72 vs. H24) and persisted into early atrophy (A24 vs. H24). However, synthesis of ribosomes was nearly absent, as evidenced by comparisons of late-stage atrophy with homeostatic conditions (A72 vs. H72) and early atrophy (A24 vs. H24) (Figure 4(B) right). Given the prevalent mitochondrial energy metabolism-related GO terms identified previously, we further investigated mitochondrial ribosomal proteins in detail (Figure 4(C)). We observed a modest increase in degradation under atrophic relative to homeostatic conditions (A24 vs. H24 and A72 vs. H72) (Figure 4(C) left). Interestingly, mitochondrial ribosomal proteins displayed reduced synthesis rates in early atrophy compared to homeostatic conditions, but synthesis notably increased over 2 days of atrophy (A72 vs. A24), surpassing the synthesis levels observed during 2 days of homeostatic state (H72 vs. H24) (Figure 4(C) right). Similar to myofibrillar proteins, ribosomal and mitochondrial ribosomal proteins exhibited an inverse pattern of regulation, with individual proteins undergoing either degradation or synthesis, but not both, across the examined conditions.

### Mitochondrial remodeling and energetic adaptation upon TNF-α-induced atrophy

Because we observed increased synthesis of mitochondrial ribosomal proteins, we assessed mitochondrial function during TNF-α-induced atrophy. We measured mitochondrial mass, abundance of mitochondrial superoxide, and ATP content. MitoTracker Green fluorescence intensity, indicative of mitochondrial abundance, was significantly reduced in atrophic myotubes (Figure S2B). MitoSOX Green fluorescence, reporting mitochondrial superoxide generation, decreased during early atrophy but shows a mild trend toward recovery after 72 h of atrophy (Figure S2C). Quantitative ATP measurements showed a marked decrease in the mean cellular ATP content from 3.1 fmol/cell at 24 h and 2.9 fmol/cell at 72 h under homeostatic conditions to 1.0 fmol/cell at 24 h of atrophy, followed by a modest rebound to 1.6 fmol/cell at 72 h of atrophy (Figure S2D), suggesting an acute energetic deficit during early atrophy and a partial recovery during late stages.

### Increased autophagic turnover upon TNF-α-induced atrophy

**Figure 5.**
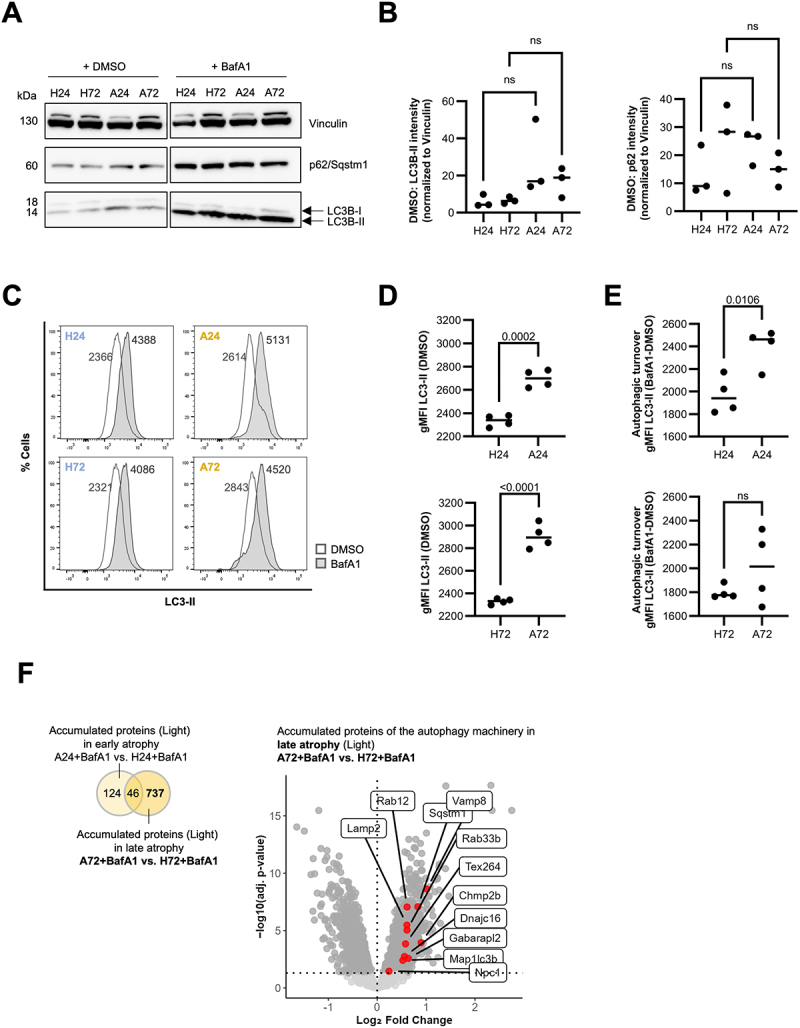
Increased autophagic turnover in TNF-α-induced atrophying C2C12 cells. (A) Western blot analysis of LC3B-II and p62 in C2C12 myotubes under homeostatic (H24, H72) and atrophic (A24, A72) conditions, with and without Bafilomycin (BafA1) treatment at time points 24 h and 72 h. Vehicle-treated (DMSO) cells serve as control. (B) LC3B-II and p62 intensities were normalized against Vinculin. *n* = 3 replicates. ns = not significant. (C-E) Flow cytometry-based assay measuring autophagic turnover by staining membrane-bound LC3-II with a fluorescently labeled antibody [30] (C) Representative histograms display the shift in LC3-II signal following 3 h BafilomycinA1 (BafA1) treatment (D) Elevated LC3-II signal intensity (gMFI, geometric mean fluorescence intensity) in atrophying (A24, A72) cells compared to homeostatic cells (H24, H72) in dimethylsulfoxide (DMSO)-treated samples. *n* = 4 replicates. ns = not significant. (E) Autophagic turnover was calculated by determining the difference in LC3-II signal intensity (gMFI) between BafA1-treated and DMSO-treated cells over a 3 h incubation period. *n* = 4 replicates. (F) Accumulated proteins upon BafilomycinA1 (BafA1) treatment in ANOVA comparisons of atrophic (A24, A72) vs. homeostatic conditions (H24, H72) in the light channel (log_2_FC > 0 adj. *p*-value < 0.05). Left: Venn diagram numbers depict significantly regulated proteins per comparison. Right: Red dots in volcano plot depict autophagy machinery components.

To assess autophagic activity under atrophying conditions, we quantified LC3-II levels in C2C12 cells using western blot and flow cytometry-based approach [30]. C2C12 cells were differentiated for 7 days, followed by 24-h or 72-h incubation with or without TNF-α to model homeostatic and atrophying conditions. To evaluate autophagic activity, cells were treated for 3 h with BafA1 (or vehicle-treated) to inhibit lysosomal function and thereby degradation of LC3B-II, causing an accumulation of LC3B-II positive autophagosomes. Western blot analysis revealed a mild, non-significant trend toward LC3B-II accumulation in DMSO-treated samples under atrophying conditions at 24 h, suggesting an increased presence of autophagosomes, potentially reflecting enhanced formation (Figure 5(A,B)). BafA1-treatment led to increased LC3B-II signal compared to DMSO-treated samples (Figure S3A), indicating functional and active autophagic activity in both homeostatic and atrophying conditions but no further accumulation was observed between homeostatic and atrophy samples within BafA1-treated samples (Figure S3B), perhaps due to signal saturation. There was also a trend toward some autophagosomal/lysosomal turnover of p62/Sqstm1 with BafA1, based on an increase in the signal after BafA1 treatment, albeit not evidently increased in atrophy (Figure S3C). To confirm and further quantify the autophagic activity, we applied flow-cytometry building on previous studies in C2C12 myotubes [31], extending it to monitor LC3-II dynamics. Flow cytometry is more quantitative and reproducible than Western blot [30] because it provides single-cell resolution, therefore not requiring normalization using loading controls, avoids signal saturation, and excludes dead cells. LC3-I was washed out with a mild detergent treatment before staining intracellularly for LC3-II only. The gating strategy is shown in Fig. S3D. Interestingly, LC3-II signal intensity significantly increased among DMSO-treated samples under atrophying compared to homeostatic conditions (Figure 5(D)), suggesting increased basal presence of autophagosomes. This was accompanied by an increased autophagic turnover at 24 h of atrophy (Figure 5(C,E), H24 vs. A24), quantified as the difference in LC3-II signal between BafA1- and DMSO-treated samples. A similar trend was observed at 72 h of atrophy, although not significant (Figure 5(C,E), H72 vs. A72). These findings suggest an increase in autophagic activity in atrophy.

To validate this, we analyzed the accumulation of autophagy machinery components in BafA1-treated atrophying conditions compared to BafA1-treated homeostatic conditions in our dynamic SILAC dataset (Figure 5(F)). Proteins with a significant increase in the light channel (log_2_FC > 0, adj. *p*-value < 0.05) were considered to accumulate as a result of inhibited autophagic turnover. Strikingly, a markedly higher number of proteins accumulated at 72 h of atrophy compared to 24 h of atrophy with 783 and 170 significantly enriched proteins, respectively, and minimal overlap (*n* = 46). In line with our flow cytometry-based analysis, Map1lc3b was among the proteins that accumulate. Additionally, other autophagy-related factors – including Lamp2, Tex264, Rab33b, and Gabarapl2 – accumulated as well.

### Autophagic receptor p62/Sqstm1 is localized at the M band of the sarcomere but lacks additional autophagic machinery

**Figure 6.**
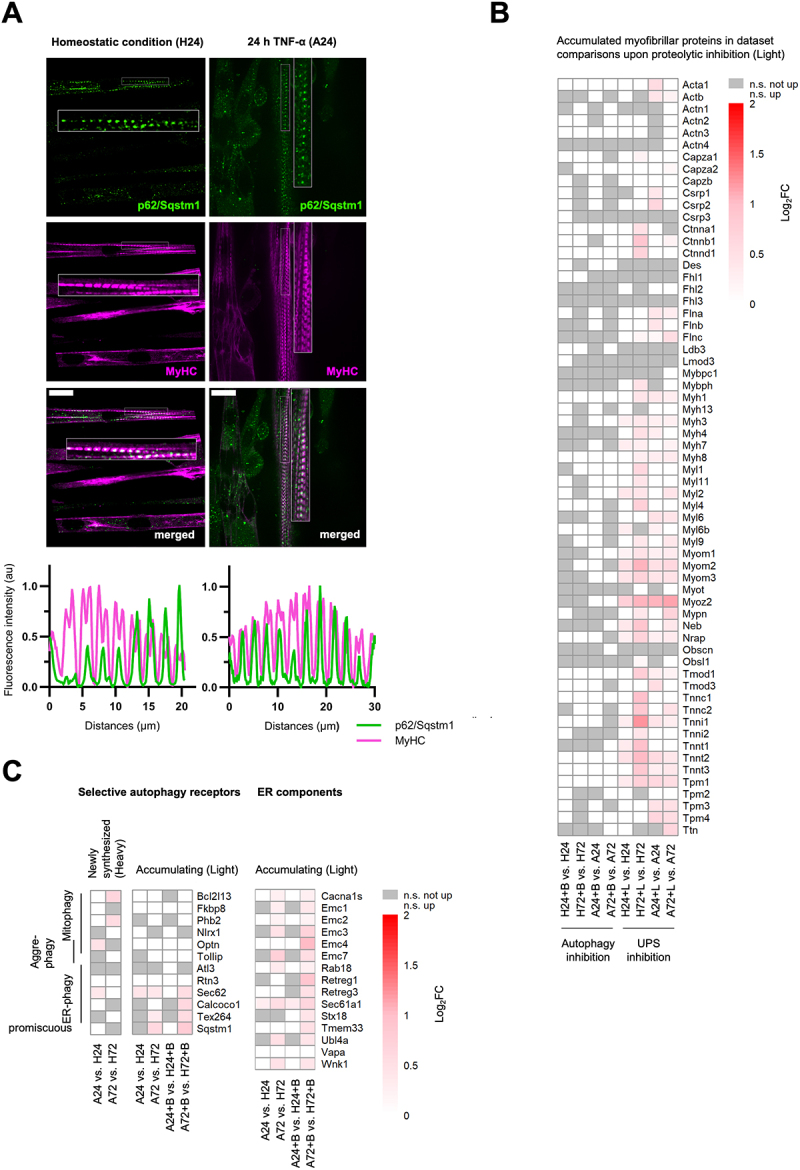
During TNF-a-induced atrophy, canonical autophagy does not mediate myofibrillar protein degradation, whereas ER-phagy emerges as the predominant form of selective autophagy. (A) Immunocytochemical staining of C2C12 (atrophying) myotubes for p62/Sqstm1 and myosin heavy chain (MyHC) highlighting the M band region of the sarcomere with corresponding line plots displaying fluorescence intensity profiles for each channel along the length of the sarcomere. Scale bar 20 µm. (B) Accumulated myofibrillar proteins upon BafilomycinA1 (+B) and Lactacystin (+L) treatment in ANOVA comparisons of homeostatic (H) and atrophying (A) C2C12 cells (light channel; log_2_FC > 0, adj. *p*-value < 0.05). White: not significantly accumulated; grey: not significant. (C) ANOVA of differentially regulated proteins (log_2_FC > 0; adj. *p*-value < 0.05). Left: Newly synthesized proteins in atrophy (A24 vs. H24 and A72 vs. H72) and accumulating (Light channel) selective autophagy receptors ± BafilomycinA1 (+B) treatment in atrophy (A24 vs. H24 and A72 vs. H72). Right: Accumulating ER components ± BafilomycinA1 (+B in atrophy (A24 vs. H24 and A72 vs. H24). White: not significantly accumulated; gray: not significant.

To gain further insight into the function of autophagy in skeletal muscles (particularly during atrophy), we aimed to locate the autophagy machinery within the sarcomere. The protein p62/Sqstm1 functions as a selective autophagy receptor, enabling it to specifically target ubiquitylated substrates for engulfment by autophagosomes, which are subsequently degraded upon fusion with lysosomes. In immunocytochemical stainings of (atrophying) C2C12 cells, we observed p62/Sqstm1 puncta formation, co-localized with myosin heavy chain (MyHC), quantified with line plots along the sarcomere (Figure 6(A)). This is in line with observations in cardiomyocytes [32,33] and indicates p62/Sqstm1 localization at the M band of the sarcomere in the skeletal muscle. This pattern was also observed with BafA1 treatment (Figure S5, S6).

However, we did not observe any strong colocalization of other autophagy machinery components like LC3B, Wipi2 (phagophore marker), lysine 63-linked ubiquitin chains (K63-Ub) and Lamp1 (lysosomal marker) with MyHC in neither C2C12 myotubes (Figure S4) nor TNF-α-induced atrophying myotubes (Figure S5), regardless of autophagy inhibition (Figure S6, S7), suggesting a lack of accumulation or functional assembly of the broader autophagy machinery in this specific region of the sarcomere.

### Inhibition of autophagy does not cause accumulation of myofibrillar protei6s

Given the presence of p62/Sqstm1 at the M band in the absence of other autophagy machinery components, we investigated whether autophagy contributes to the degradation of myofibrillar proteins. To assess the influence of either proteolytic system, i.e. autophagy and UPS, on the degradation of myofibrillar proteins, proteolytically inhibited and non-inhibited samples were compared using the preexisting protein pool (Light channel) (Figure 6(B)). Proteins showing a significant increase in the light channel (log_2_FC > 0, adj. *p*-value < 0.05) were classified as accumulated upon inhibition. Inhibition of the UPS (+L, Lactacystin) led to significant accumulation of numerous myofibrillar proteins from all regions of the sarcomere, in both homeostatic and atrophying myotubes, with the strongest accumulation observed in homeostatic conditions (H72+Lac vs. H72). Conversely, the inhibition of autophagy (+B, BafilomycinA1) did not result in significant accumulation of myofibrillar proteins, suggesting minimal or no involvement of autophagy in their degradation.

### Shift in autophagic selectivity from homeostatic ER and metabolic maintenance to ER-stress-linked degradation in TNF-α-induced atrophy

To further characterize the autophagic cargo selectively targeted during atrophy compared to homeostatic conditions, we performed GO analyses on proteins accumulating upon autophagy inhibition in both conditions (Figure S8, S9). Again, proteins showing a significant increase in the light channel (log_2_FC > 0, adj. *p*-value < 0.05) were classified as accumulated upon autophagic inhibition. Under homeostatic conditions (H+BafA1 vs. H), 73 proteins accumulated compared to 91 accumulating in atrophic conditions (A+BafA1 vs A) upon autophagy inhibition with an overlap of 38 proteins in both conditions (Figure S8A). Both homeostatic and atrophying conditions share a core set of enriched terms upon inhibition, including ECM-related processes, cytoskeletal components, and ER-localized multiprotein complexes. However, proteins accumulating in atrophy were additionally associated with protein folding, maturation, and ER stress response (like Hspa5/BiP) (Figure S8B), whereas proteins accumulating in homeostatic conditions were involved in metabolic processes such as alcohol and cholesterol metabolism and cellular response to nitrogen compounds (Figure S8B). Furthermore, upon autophagy inhibition in atrophic conditions, more BCAA transporters were found to be accumulated than in homeostatic conditions (e.g. Slc3a2 in early and Slc7a5/Lat1 in late atrophy). To conclude, while autophagy inhibition in homeostatic conditions led to accumulation of proteins related to metabolic processes and ER quality control, autophagy inhibition in atrophying condition led to accumulation of proteins related to ER quality control as well as ER stress – indicating a shift in autophagic selectivity during atrophy.

We next distinguished early and late atrophic conditions by analyzing GO terms associated with proteins accumulating upon BafA1 treatment in early (A24+BafA1 vs. A24) and late atrophy (A72+BafA1 vs. A72), respectively (Figure S9A-B). A total of 65 proteins accumulated in early atrophy, while 56 proteins accumulated in late atrophy, with 30 shared hits between both conditions. Both early and late atrophy showed a strong enrichment for extracellular matrix (ECM)-related processes (e.g. Col1a2, Fn1), ECM-receptor interaction, vasculature development and cytoskeleton-related processes (Figure S6B). Similarly, amyloid-beta clearance (e.g. Lrp1, Lrp4) and regulation of nervous system development (e.g. Ephb3, App) were enriched in both early and late atrophy. Importantly, proteins related to protein folding (e.g. Pdia4, Hsp90b1) and ER-resident proteins (e.g. Tor1a, Nomo1) were accumulated in both states. Nevertheless, the proteome accumulating with BafA1 during early atrophy showed more hits related to an ER stress response (e.g. Pdia3, Hspa5, Dnajc3), the ER-associated degradation (ERAD) pathway and the UPR (proteasome subunits). In addition, BafA1 treatment in late atrophy led to the accumulation of more autophagosome-related hits compared to early atrophy (Figure S6A): Gabarapl2, Map1lc3a, Stx12, Rab11b and also to a higher accumulation of autophagy receptors Calcoco1 and p62/Sqstm1, indicating increased abundance of autophagosomes as it is expected after BafA1 treatment and elevated autophagic activity in atrophy. These findings are consistent with an emphasis on ER-phagy during atrophy.

### ER-phagy is the most prominent selective autophagy type during TNF-α-induced atrophy

**Figure 7.**
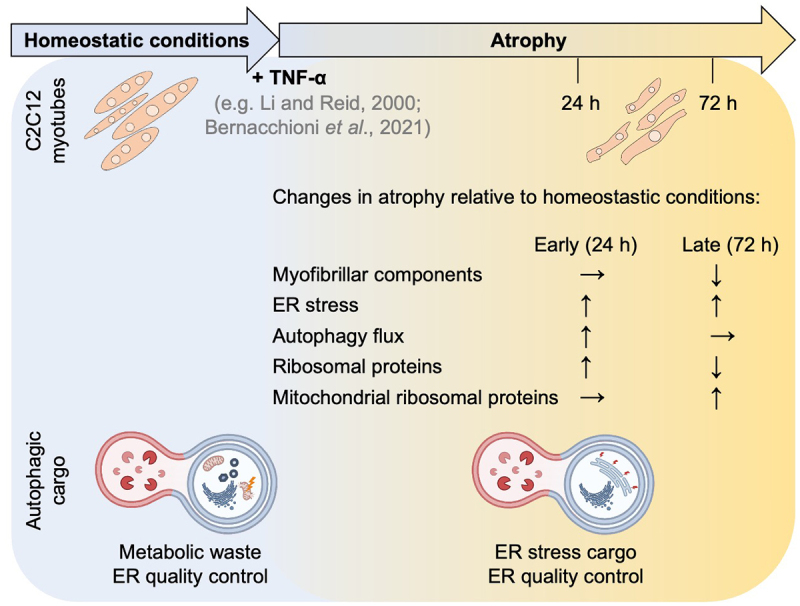
Schematic model of proteostatic remodeling in TNF-α-induced muscle atrophy over time (24 h to 72 h): In C2C12 myotubes, TNF-α triggers selective remodeling of proteostasis, translation (Ribosomal and mitochondrial ribosomal proteins), ER stress, autophagic flux and autophagic cargo. Arrows represent changes observed under TNF-α-induced atrophy relative to the corresponding baseline control.

To further elucidate the specificity of autophagic response in muscle atrophy, we investigated which selective autophagy pathways are engaged or disrupted during early and late stages, respectively (Figure 6(C) left). Proteins showing a significant increase in the heavy channel (log_2_FC > 0, adj. *p*-value < 0.05) were classified as significantly newly synthesized. Among all published selective autophagy receptors [34], we observed minimal new synthesis of selective autophagy receptors comparing early atrophy to homeostatic conditions (A24 vs. H24), only Optn (mitophagy/aggrephagy) and Sec62 (ER-phagy) increased indicating a response to unfolded proteins and ER stress (Figure 6(C) left). Interestingly, in late-stage atrophy (A72 vs. H72), two mitophagy receptors (Bcl2l13 and Phb2) were newly synthesized but no ER-phagy receptors were synthesized (Figure 6(C) left). Autophagy receptors are known to accumulate upon BafA1 treatment due to impaired lysosomal degradation. To further explore this, we examined which receptors accumulate significantly in atrophying conditions compared to homeostatic conditions upon autophagy inhibition (light channel, log_2_FC > 0, adj. *p*-value < 0.05) (Figure 6(C) left). Interestingly, in early atrophy, none of the monitored autophagy receptors accumulated, suggesting functional autophagic turnover. In contrast, we detected an accumulation of three ER-phagy receptors, Sec62, Calcoco1 and Tex264, as well as p62/Sqstm1 with or without BafA1 treatment in atrophy. To further investigate ER-phagy dynamics, we monitored ER-resident proteins and found that while early atrophy showed negligible accumulation, late atrophy resulted in a marked accumulation of ER components (Figure 6(C) right). Collectively, these data indicate that autophagy is degrading ER, reaching its peak at 72 h of atrophy. To validate the proteomic indication of ER stress during atrophy, we used a stable C2C12 cell line expressing GFP-KDEL (an ER retention signal) and observed preserved ER network morphology after 24 h of TNF-α treatment, whereas at 72 h of atrophy, the GFP-KDEL signal was redistributed into distinct puncta (Figure S10A). To quantify these observations and differentiate between granular-cytosolic and distinct puncta, images were analyzed by selecting a region of interest (ROI), applying a fixed intensity threshold (20–255), and counting particles with an area of 1-9 µm^2^ (Figure S10B). To normalize for variable cell size and selected ROIs, the percentage of area occupied by GFP-KDEL-positive puncta was calculated and showed an increase upon 72 h of atrophy. We next assessed ER-phagy using ER-Tracker Green (BODIPY-glibenclamide) by flow cytometry (Figure S10C). ER-Tracker fluorescence reflects dye accessibility to ER compartment. ER signal remained stable between homeostatic 24-h and 72-h conditions. In contrast, TNF-α treatment showed a trend toward a reduction in ER signal at both 24 h and 72 h of atrophy. BafA1 treatment increased ER signal across all conditions, indicating that ER material is targeted to the autophagy-lysosome pathway in both homeostatic conditions and atrophy. Figure 7 summarizes the integrated outcome of our analyses, highlighting the early TNF-α-induced shifts in protein synthesis and degradation, the distinct temporal clustering of translationally regulated proteins, and the specific autophagy-dependent turnover patterns that were observed in the acute atrophy response.

## Discussion

This study presents a, time-resolved analysis of protein turnover in TNF-α-induced muscle atrophy via dynamic SILAC, refining the conventional view that atrophy is always accompanied by decreased protein synthesis and increased protein degradation [1,2]. Instead, our findings reveal a selective remodeling of protein dynamics across distinct protein groups, indicating an adaptive rather than purely degenerative process [35]. This aligns with previous studies showing that different atrophic and hypertrophic stimuli elicit varied responses in synthesis and degradation, such as denervation of the rat diaphragm, which leads to both increased protein degradation and enhanced protein synthesis [6]. This transition is marked by a shift in autophagic selectivity – moving from maintenance of basal proteostasis toward targeted degradation of ER components, likely mediated by specific ER-phagy receptors. Such receptor-specific recruitment of autophagy machinery underscores the potential of organelle-selectivity autophagy in atrophic remodeling.

We employed OPP labeling and dynamic SILAC to provide a snapshot of short-term translation rates (30 min) and long-term protein turnover dynamics, respectively. The combination of these methods offers complementary insights. The OPP data confirmed that translation is acutely downregulated in TNF-α-treated cells, reflecting rapid suppression of mRNA translation. However, dynamic SILAC revealed that translation suppression is not uniform. While overall synthesis rates declined and cytosolic ribosomal proteins are degraded in atrophying conditions, mitochondrial proteins continued or even increased synthesis in late atrophy. This suggests that skeletal muscle cells selectively maintain the production of essential proteins to sustain metabolic function. Considering ATP’s regulatory role in protein synthesis [36], short-term translation suppression may serve as an adaptive response saving ATP under catabolic conditions. Ribosomal dynamics further evolved over time: early atrophy showed cytoplasmic ribosome degradation and possible compensatory ribosome biogenesis, likely in response to ROS production [37]. In late atrophy, cytoplasmic ribosome synthesis stopped, potentially as an energy-saving strategy. Notably, BafA1 treatment did not lead to an accumulation of known ribophagy receptors, suggesting alternative ribosome degradation mechanisms. Dynamic SILAC analysis revealed increased synthesis of mitochondrial ribosomal proteins during atrophy, indicating activation of a compensatory translational program. However, functional assays demonstrated a concomitant loss of mitochondrial content, reduction in mitochondrial ROS, and decline in ATP levels in atrophy, suggesting that enhanced mitochondrial protein synthesis reflects an attempted remodeling or repair response of aberrant mitochondria rather than a net increase in mitochondrial content. Mitochondrial remodeling in atrophy potentially supports OXPHOS and ATP production under catabolic stress, consistent with mitochondrial dysfunction reported in disuse atrophy [38].

Under normal conditions, misfolded proteins are shuttled to the ER and degraded by the proteasome via ERAD. When ERAD capacity is exceeded, UPR activation attenuates translation [39], promotes autophagic degradation [40] and increases chaperone expression [41]. Prolonged ER stress ultimately leads to a decline in protein folding capacity, exacerbating proteostasis disruptions and muscle damage [42]. TNF-α has been shown to induce the UPR [43], and ATF4 activation in muscle atrophy models [44] supporting the link between UPR and autophagy-mediated muscle atrophy. The chaperone Hspa5/BiP, crucial for ER protein folding, thereby maintaining ER homeostasis, accumulated upon BafA1 treatment in early atrophy compared to homeostatic state, indicating its autophagic degradation early in atrophy. While Hspa5/BiP is typically targeted for degradation via the UPS [45], we propose autophagic degradation under TNF-α-induced atrophy under severe or prolonged ER stress. Autophagic degradation of ER chaperones like Hspa5/BiP has been observed before upon proteasomal inhibition in *in vitro* studies by [46]. While early atrophy induced synthesis of receptors like Optn (aggrephagy, mitophagy) and Sec62 (ER-phagy), no ER-phagy receptors were newly synthesized at 72 h. However, during late atrophy, we could observe significant accumulation of ER-phagy receptors (Sec62, Calcoco1, and Tex264), which are understudied ER-phagy receptors in the muscle context as well as p62/Sqstm1 which acts as a promiscuous receptor in both canonical and non-canonical autophagy. The coordinated accumulation of these receptors suggests a temporally regulated, receptor-guided engagement of ER-phagy. This adds to a growing recognition that autophagy is not merely a bulk process but involves cargo-selective mechanisms with specialized receptors modulating the finely tuned degradation of distinct targets during atrophic stress. Further supporting a progressive shift in proteostasis, protein folding pathways became increasingly downregulated in late atrophy. We could observe that TNF-α-induced atrophy shifts autophagic selectivity from maintaining homeostatic balance toward increased degradation of ER components upon atrophy stimulus. Complementing these proteomic signatures of ER stress and enhanced ER-phagy engagement, our ER morphology and ER-Tracker assays indicate progressive ER remodeling during atrophy.

Patients suffering from Duchenne muscular dystrophy (DMD) – a muscular disease caused by the loss of dystrophin [47] – eventually develop muscle atrophy due to a combination of interconnected factors. Chronic inflammation [48] contributes to fibrosis, increased susceptibility to mechanical damage [49] and impaired regeneration [50], all of which progressively lead to muscle degeneration. DMD patients show an activation of the UPR and increased abundance of Hspa5/BiP [51–53] and a zebrafish DMD KO model with an Hspa5/BiP inhibitor improved muscle function [54]. Ruparelia *et al*. have also recently shown in zebrafish skeletal muscle that upon loss of Atrogin-1, a well-studied upregulated E3 ligase under atrophying conditions [2,5] not only Hspa5/BiP accumulates but also leads to downregulation of autophagic flux in skeletal muscle impaired mitochondrial dynamics and loss of skeletal muscle fiber structure [55]. Upregulation of ER-stress/UPR-related factors and Hspa5/BiP was studied in a number of other muscle diseases like tibial muscular dystrophy caused by mutations in titin with altered autophagy levels [56]. The progressive increase in ER degradation after TNF-α-induced atrophy observed in our dynamic SILAC data aligns with findings from Atg7 muscle knockout models where atrophy is exacerbated, accompanied by dilated sarcoplasmic reticulum (SR)/ER structures and observed upregulation of Hspa5/BiP, indicating activation of the UPR [22].

Early atrophy involves upregulation of ubiquitin ligase activity, neddylation, and proteasomal function, indicating UPS activation. This is supported by myofibrillar accumulation upon UPS inhibition. Late atrophic phase is marked by a pronounced myofibrillar degradation, consistent with previous reports [57], but also degradation of ECM and cytoskeletal components. This occurs alongside a decline in proteasome activity and neddylation, as indicated by GO terms associated with the significantly degraded proteins, suggesting a progressive impairment of the UPS over time. Despite increased autophagic turnover in atrophy, autophagy minimally contributes to myofibrillar degradation, as seen in the lack of significant accumulation of myofibrillar components upon BafA1 treatment. Furthermore, no spatial localization of the autophagy machinery to the M band was observed, even in the presence of p62/Sqstm1. Based on the significant accumulation of sarcomeric components solely upon UPS inhibition, we propose the UPS as the dominant mediator of myofibrillar protein degradation. The mechanistic basis for its selectivity over autophagy remains to be clarified. It is plausible that myofibrillar proteins require preliminary disassembly, e.g. via calpains, but their involvement in sarcomeric degradation remains incompletely understood and requires further investigation. The structural organization of sarcomeres may restrict their engulfment by autophagosomes. Another consideration is that autophagy may primarily target organelles and soluble proteins, rather than large, insoluble protein complexes like myofibrils. Whether or not that differs between fiber types is beyond the scope of this study but highlights an important area for future investigation.

Non-canonical functions for p62/Sqstm1 have been described before, linking it to activation of the Keap1-Nrf2 pathway for antioxidant response [58,59], activation of mTORC1 in nutrient sensing [60] and activation of NF-κB during apoptosis and inflammation [61]. However, even under autophagy-inhibited conditions, other core autophagy components did not specifically localize to the M band, excluding the possibility that the absence of autophagy markers was due to rapid vesicle turnover, supporting a non-canonical role of p62 at this site.

The SR acts as a specialized form of the ER in skeletal muscle, regulating calcium storage and release for muscle contraction [62,63]. Despite its distinct function, it retains core ER properties, including protein folding and processing. The fiber-type transition associated with inflammation-induced atrophy, from fast-twitch (glycolytic) to slow-twitch (oxidative) fibers [64,65] may necessitate structural SR remodeling, given that glycolytic fibers have a more extensive SR network for rapid calcium cycling [66], while oxidative fibers rely more on mitochondrial ATP production. This aligns with our observation that mitochondrial ribosomal proteins are newly synthesized in atrophy, suggesting that muscle cells shift toward mitochondrial respiration phenotype as part of their adaptive response. Similar SR remodeling has been observed during exercise adaptation [66]. Our results hint toward a highly regulated form of SR/ER remodeling via elevated autophagic activity beyond UPR during TNF-α-induced muscle atrophy.

Autophagy plays an essential role in maintaining muscle proteostasis by clearing damaged organelles and misfolded proteins, thereby mitigating ROS, alleviating ER stress and recycling metabolites. In this regard, moderate activation of autophagic flux has been shown to preserve muscle integrity. For example, the reactivation of autophagy through Atg7 re-expression or pharmacological inducers such as Urolithin A enhances mitophagy, improves mitochondrial quality, and alleviates muscle dysfunction in models of sarcopenia [67]. In contrast, excessive or dysregulated autophagy can contribute to muscle wasting. Muscle-specific Atg7 deletion causes mitochondrial dysfunction and sarcomeric disarray [22], underscoring the need for balanced autophagy. Likewise, the overexpression of FOXO3, an upstream driver of autophagy genes, induces excessive autophagic flux and results in pronounced muscle wasting [68]. Loss-of-function mutations in *Jumpy*, a negative regulator of autophagy initiation, leads to centronuclear myopathy due to autophagic activation [24]. Additional knockout models targeting Atg5, mTOR, ULK2, VPS15, and AMPK further emphasize the finely tuned regulation required for autophagy to remain beneficial [69–73]. These studies highlight that the impact of autophagy on muscle health is highly context-dependent: adaptive under moderate activation – supporting proteostasis and metabolic resilience – and maladaptive when excessively induced or long-term dysregulated. Our findings on ER-phagy fit within this framework, suggesting that its role during TNF-α-induced atrophy reflects a context-dependent adaptation to stress that, when prolonged, may transition toward a maladaptive state.

While our dynamic SILAC study uses a simplified model of TNF-α-induced atrophy in C2C12 cells over a 72-h period, it enables precise temporal mapping of early changes in protein turnover and autophagy under defined inflammatory stress. Although it does not mimic the full complexity of chronic muscle-wasting diseases, it captures early proteostatic remodeling events relevant to acute conditions such as sepsis where degeneration is rapid. It offers mechanistic insights into the initial cellular responses that may initiate or shape both acute and longer-term atrophic progression. We acknowledge that TNF-α not only promotes atrophy signaling but can also activate autophagy directly, through NF-κB-dependent pathways [12]. Rather than viewing this as a confounding effect, we consider it physiologically relevant, as inflammatory cytokines *in vivo* elicit parallel activation of catabolic and autophagic programs that jointly shape muscle remodeling [74]. The ER-phagy signatures we observe may therefore reflect a broader, integrated stress response that coordinates degradation of misfolded proteins and ER turnover during inflammatory challenges. Importantly, *in vivo* studies have demonstrated that autophagy activation under septic conditions plays a protective role in muscle tissue [23] reinforcing the translational relevance of our *in vitro* findings. Since our study focuses on TNF-α as the sole atrophy trigger, future studies should test whether the temporal patterns of ER-phagy identified here extend to more complex cytokine milieus (IL-1β, IL-6, IFN-γ) that better model systemic inflammation.

While C2C12 myotubes offer a well-controlled platform to dissect the temporal dynamics of proteostasis, they lack several key features of skeletal muscle physiology, including fiber-type heterogeneity, satellite cell interactions, innervation, vascularization, and systemic hormonal or immune cues. As such, our findings primarily reflect molecular events in a homogeneous *in vitro* myotube population and may not fully capture the regulation of proteostasis *in vivo*. The observed relationships between autophagy inhibition, ER-phagy receptor accumulation, and atrophic remodeling suggest a coordinated remodeling of selective autophagy pathways, though direct causal relationships will require further validation. Future studies are planned to extend these findings to primary myotubes, *ex vivo* muscle fibers, and animal models of acute and chronic muscle atrophy, as well as to employ autophagosome pulldown assays to define autophagic cargo selectivity. Moreover, induction of ER stress and UPR activation, combined with high-resolution morphological analyses of ER structure, will help elucidate how inflammation-driven ER remodeling integrates with selective autophagy during muscle atrophy.

## Materials and methods

### Cell culture

WT C2C12 mouse skeletal muscle myoblasts (ATCC, CRL-1772) and ssGFP-KDEL C2C12 cells (kindly provided and originally described by Buonomo *et al*. [75]) were grown adherently and undifferentiated in DMEM (Sigma-Aldrich, D5796) supplemented with 10% heat inactivated fetal bovine serum (FBS) (Gibco, 10500-064) and 1% Pen/Strep (P/S) (Sigma-Aldrich, P4333). Myogenic differentiation was initiated upon reaching confluence by switching the cells to a medium containing 2% FBS and kept in culture for 7 to 10 days, replenishing the medium every second day. Atrophy was induced on the seventh day with 10 ng/ml tumor necrosis factor α (TNF-α) (Thermo Fisher, RMTNFAI) and cells cultivated for up to 72 h. For dynamic SILAC studies, WT C2C12 cells were cultured in SILAC medium (PANBiotech, P04-02505) with 10% dialyzed FBS (Merck, F0392) and 200 mM GlutamaxTM (Thermo Fisher, 35050061). Cells were differentiated for 7 to 10 days in light SILAC medium (PANBiotech, P04-02505) supplemented with 200 mM GlutamaxTM (Thermo Fisher, 35050061), 2% FBS, P/S (Gibco, 15140-122) and 28 mg/L light arginine 0 (R0 = Arginine 0 [^12^C_6_, ^14^N_4_], Sigma, A6969) and 48.7 mg/L light lysine (K0 = Lysine 0 [^12^C_6_, ^14^N_2_] Sigma, L8662). For pulsing with heavy medium, Arg0 and Lys0 in SILAC medium were replaced with 34 mg/l heavy arginine and 50.7 mg/l lysine (R10 = Arginine 10 [^13^C_6_, ^15^N_4_], Silantes 211604302 LOT (211CXN-LysI-503-02); K8 = Lysine 8 [^13^C_6_, ^15^N_2_], Silantes 201604102 LOT (201CXN-ArgI-603-01). ss-GFP-KDEL cells were used for imaging of the ER (GFP-KDEL), Golgi (GM130), and nuclei (DAPI) as described below.

### Immunocytochemical (ICC) staining

Cells were cultured on ibidi polymer coverslips (Ibidi 81156), fixed in 4% paraformaldehyde (PFA) and permeabilized with DPBS (Gibco, 14040-117) containing 0.4% Triton X-100. Proteins were stained with the following commercial antibodies: rat monoclonal anti-LAMP1-FITC (BioLegend, sc-121606 1:1000), rabbit polyclonal anti-LC3B (Novus Biologicals, NB100-2220 1:500), mouse monoclonal anti-MyHC (Sigma-Aldrich, M4276 1:1000), rabbit polyclonal anti-p62 (Invitrogen, PA5-20839 1:1000), rabbit monoclonal anti-Ub K63 (Sigma-Aldrich, 05-1308 1:1000), rabbit polyclonal anti-WIPI2 (Atlas Antibodies, HPA019852 1:400), DAPI (Thermo Fisher, 62249) goat anti-mouse IgG (H+L) secondary antibody AF647 (Thermo Fisher, A-21235) and goat anti-rabbit IgG (H+L) secondary antibody AF488 (Invitrogen, 10729174). To distinguish discrete ER-associated puncta from the granular cytosolic GFP-KDEL signal in ssGFP-KDEL C2C12 cells, puncta were quantified using ImageJ. For each condition, five individual cells were selected as regions of interest (ROIs). A fixed intensity threshold (20–255, minimum-maximum gray values) was applied uniformly to all images. The resulting binary masks were analyzed with the Analyze Particles function, and puncta were counted if their area ranged from 1 to 9 µm^2^. The total number of puncta per cell was recorded for further analysis, and statistical differences between conditions were assessed using a one-way ANOVA followed by Tukey’s multiple-comparison test. Cells were imaged on a Nikon Ti inverted microscope equipped with a Yokogawa CSU-W1 spinning disk confocal scanner, a Plan Apo λ 100x/1.45 NA oil-immersion objective and an Andor-DU-888 EMCCD camera. Fluorophores were excited using 488 nm and 647 nm laser lines. Images were acquired at 1024 × 1024 resolution. Z-stacks were acquired with step sizes ranging from 0.2 to 0.5 µm depending on the sample and region of interest. All image acquisition settings (exposure time, laser power, camera gain) were kept constant across conditions. Image processing was performed using Fiji (ImageJ 2.9.0), with only linear adjustments to brightness and contrast applied equally to all images.

### O-propargyl-puromycin (OPP) assay

For OPP labeling experiments, differentiated and atrophying C2C12 myotubes were incubated with 20 µM OPP (Thermo Fisher, C10459) for 30 min in growth medium at 37°C in the incubator and then fixed and permeabilized. Cycloheximide-treated samples served as negative control. The OPP-incorporated proteins were then fluorescently labeled via a copper-catalyzed click reaction and immediately analyzed via confocal microscopy. OPP puncta were manually counted across 20 images, each containing over 200 cells, with statistical significance assessed using ANOVA and Tukey’s test.

### Pharmacological inhibitors

To reduce the frequency of undifferentiated myoblasts in C2C12 cell populations which were subjected to differentiation, cells were treated with 50 µM Cytosin-1-β-D-arabinofuranosid (AraC, Sigma Aldrich, C1768) on day 4 of differentiation for 72 h. To inhibit proteolytic processes, either 50 µM Lactacystin (UPS inhibitor, Adoq Bioscience, A12768) or 50 nM Bafilomycin A1 (autophagy/lysosome inhibitor, Sigma Aldrich, B1793) was applied 3 h prior cell harvest. For translation inhibition, 50 µg/ml cycloheximide (Fluka BioChemika, 01811) was applied 90 min prior cell harvest or subsequent treatment.

### Automated protein processing and digestion for mass spectrometry

C2C12 cells were harvested, lysed in 50 mM Tris-HCl with 0.5% SDS and protease inhibitor (Roche, 04693132001) and protein concentrations were determined using a BCA assay (Thermo Fisher Scientific, 23250). Ten micrograms of each sample (100 µl) were transferred to a 96-well Armadillo plate and processed on an OT-2 robot (Opentrons). Proteins were reduced with 10 mM DTT at 37°C, 1000 rpm for 30 min, followed by alkylation with 15 mM iodoacetamide under the same conditions. The reaction was quenched with 30 mM DTT. Magnetic Sera-Mag SpeedBeads (1:20 bead-to-sample ratio) were added and incubated for 5 min at 1000 rpm. Proteins were bound with 100 µl acetonitrile, then washed sequentially with 80% ethanol and acetonitrile. Digestion was performed overnight at 37°C, 1150 rpm using LysC and trypsin (1:50 enzyme-to-protein ratio) in 100 mM ammonium bicarbonate. Peptides were collected into a new 96-well plate, dried via vacuum centrifugation and resuspended in Buffer A for LC-MS analysis.

### Mass spectrometry data acquisition

Each sample (1 µg) was analyzed using an Exploris 480 mass spectrometer coupled to a Vanquish Neo UHPLC (Thermo Fisher) with a 106-min nanoflow (0.25 µl/min) gradient. Peptides were separated on a 20-cm, 1.9-µm in-house packed column. The gradient ramped from 2% to 30% of a buffer (0.1% formic acid, 90% acetonitrile) over 88 min, followed by 60% for 10 min, and 90% for 5 min. MS1 scans (350–1650 m/z) were acquired at 120,000 resolution; MS2 scans used 40 isolation windows with a maximum injection time of 54 ms. The method was adapted from [76].

### Mass spectrometry data processing and analysis

Raw data were processed using DIA-NN (v1.8.1) with a project-specific spectral library generated from the *Mus musculus* UniProt FASTA database (UP000000589). SILAC-specific settings were applied (see Supplementary Methods for full parameters). The analysis pipeline from [77] was adapted to handle dynamic SILAC experiments without relying on an internal heavy reference. First, contaminants were removed, before filtering out SILAC precursors where either H or L precursors failed to meet the following criteria: Global.PG.Q.Value < 0.01, Precursor.Charge > 1, and Channel.Q.Value < 0.03. Intensity values (MS1.Translated and Precursor.Translated) were used to calculate SILAC precursor ratios, the median log2 value of which was assigned to protein group-level ratios within each run. For cases where only H or L precursors were present per protein group, a single-channel mask was applied, meaning the total abundance was allocated entirely to either H or L. The total intensity was computed by summing the H and L Precursor. Quantity values of each SILAC pair together, then passing these total intensity values to directLFQ for normalization [78]. The total abundances after normalization were then split into H and L datasets based on protein group ratios or the single-channel mask, producing normalized newly synthesized and aging proteomes, respectively, suitable for cross-sample comparison. An updated version of the code used for this analysis was implemented in a standalone package: https://github.com/rkerrid/StackedLFQ.

### Statistical analysis

Quantitative proteomics data from light channels, heavy-labeled peptides, and total signals were processed and analyzed in R (v4.5.0) using the limma package (v3.65.1) [29] or all datasets, protein intensities were log2-transformed, and normalization by median centering was applied (applicable to heavy-labeled data only). Proteins were retained for analysis if they contained at least three non-missing values per experimental group (applicable to heavy-labeled and total signal datasets). For each protein, a linear model was fitted, incorporating an intercept and a categorical factor representing the experimental groups. Empirical Bayes variance moderation was applied, with robust and trend estimation enabled. Differential abundance was assessed using a moderated global F-test to jointly evaluate all non-intercept coefficients, equivalent to an omnibus test for group differences. Resulting *p*-values were adjusted for multiple testing using the Benjamini–Hochberg procedure. Proteins with an adjusted *p*-value below 0.05 were considered statistically significant.

Figure 1(C): Calculation of intensity clusters: Normalized light and heavy intensities were summed to calculate the total intensities of individual proteins at time points t0, t24, and t72 to create temporal profile clusters (Figure 1(C)). Cluster 2 was calculated as follows: constant intensity from t0 to t72, defined as exhibiting no more than a 10% variation relative to the maximum change intensity (Δ_max, with the 10% threshold calculated as 0.1 × Δ_max).

### Data availability

All raw mass spectrometry data and processed Excel spreadsheets containing significantly deregulated proteins (including ANOVA comparisons of both heavy and light fractions) are available in the following Zenodo repositories: DOI: 10.5281/zenodo.15915562 and DOI: 10.5281/zenodo.15916917.

### Gene Ontology (GO) enrichment analysis

GO enrichment analysis (biological terms) was performed using Metascape to identify significantly overrepresented biological processes among differentially expressed proteins [79].

### Western blot analysis

Cells were washed and lysed in buffer (1% Triton X-100, 10 mM ß-glycerol phosphate, 10 mM sodium pyrophosphate, 4 mM EDTA, 40 mM HEPES at pH 7.4) supplemented with protease and phosphatase inhibitors (Cell Signaling Technology, 5872) for 30 min on ice. Lysates were cleared by centrifugation at 12,700 × g for 5 min at 4°C, and protein concentrations were determined using the BCA assay (Thermo Fisher, 23235). Ten micrograms of protein was separated by SDS-PAGE on 4-20% polyacrylamide gels (Thermo Fisher, XP04205BOX) and transferred onto PVDF membranes (Bio-Rad). Membranes were blocked in 5% nonfat milk in TBS-T for 45 min and probed overnight at 4°C with primary antibodies against LC3B, p62/Sqstm1, and Vinculin (all Cell Signaling Technology, 3868S, 39749S, 13901S; all 1:1000). After washing, the membranes were incubated with HRP-conjugated secondary antibody (Vector Laboratories, PI-1000, 1:3000 in 5% nonfat milk) for 1 h at 4°C. Blots were developed using an ECL substrate (Bio-Rad, 170-5061) and imaged using a ChemiDoc system (Bio-Rad). Band intensities were quantified, and LC3B-II and p62/Sqstm1 levels were normalized to Vinculin.

### Quantification of LC3 and organelle markers using flow cytometry

C2C12 myotubes were differentiated for 7 days. On day 7, atrophy was induced by addition of 10 ng/ml TNF-α for 24 h or 72 h. Cells were harvested and washed three times with PBS. For flow cytometry analysis, cells were first stained with live/dead dye (Zombie NIR^ly^ Fixable Viability Kit, Biolegend # 423 , 105). Afterward , the Guava Autophagy LC3 antibody-based assay (Cytek # FCCH100171) was used to stain for LC3. Cells were permeabilized with PBS and 0.05% Saponin and cytosolic LC3 (LC3-I) was washed out before incubating with anti-LC3 antibody to only detect LC3-II. After the staining, cells were fixed with 4% PFA. For quantification of membrane-bound LC3-II, the geometric mean of the staining was quantified on live singlet cells. To assess organelle dynamics under atrophying conditions, complementary flow cytometry assays were performed according to the manufacturer’s protocols. ER state was quantified using ER-Tracker™ Green (BODIPY-glibenclamide) (Thermo Fisher # E34251). Mitochondrial abundance was assessed with MitoTracker™ Green (Thermo Fisher # M46750). MitoSOX™ Green (Thermo Fisher # M36005) staining was used to measure mitochondrial superoxide levels. For data analyses, we used the median fluorescence intensity (MFI) of the respective dyes from singlet live cells. Data were acquired on the same LSRFortessa™ cytometer , and analyzed using FlowJo v10.10.0.

### ATP determination assay

ATP levels in C2C12 myotubes were quantified using the ATP Determination Kit (A22066, Thermo Fisher Scientific, Waltham, MA, USA) according to the manufacturer’s instructions, with four biological replicates. Briefly, cells were lysed, and the resulting supernatants were incubated with recombinant firefly luciferase and its substrate, D-luciferin. Because luciferase catalyzes the oxidation of luciferin in a reaction strictly dependent on ATP, the emitted luminescence is directly proportional to ATP concentration. A standard curve of ATP (1 nM–5 µM) was generated in parallel in each assay plate to ensure linearity. Raw luminescence values were background-subtracted and converted to ATP concentration using the standard curve equation. ATP quantities were normalized to cell number per well, allowing estimation of ATP per cell (mol/cell). Measurements were performed in quadruplicates per condition.

## Abbreviations:

A24, A72: Atrophying conditions at time point 24 and 72 hours post-induction
Adj. *p*-value: Adjusted *p*-value
ANOVA: Analysis of variance
Autophagy: Autophagy/lysosome pathway
BafA1: Bafilomycin A1 (autophagy/lysosome pathway inhibitor)
Ca^2+^: Calcium ion
ECM: Extracellular matrix
ER: Endoplasmic reticulum
FBS: Fetal bovine serum
GO: Gene Ontology
H24, H72: Homeostastic conditions at time point 24 hours and 72 hours
ICC: Immunocytochemical
Lac: Lactacystin (proteasome inhibitor)
LC-MS/MS: Liquid chromatography – tandem mass spectrometry
Log_2_FC: Log2 fold change
OPP: O-propargyl-puromycin
OXPHOS: Oxidative phosphorylation
SILAC: Stable isotope labeling of amino acids in cell culture
SR: Sarcoplasmic reticulum
t0, t24, t72: Timepoints 24, 24 and 72 hours
TNF-α: Tumor necrosis factor alpha
UPR: Unfolded protein response
UPS: Ubiquitin-proteasome system
Vs.: Versus

## Supplementary Material

Supplementary_text_2026_final.docx

## Data Availability

The raw data from the dynamic SILAC experiments supporting this study are openly available on Zenodo at 10.5281/zenodo.15915562 and 10.5281/zenodo.15916917. Additional materials and methodological details are available upon reasonable request from the corresponding author.
