# Supplementary material for: Autophagy selectively clears ER in TNF-α-induced muscle atrophy
Source: Autophagy Rep. 2026 Apr 14;5(1):2649064. doi: 10.1080/27694127.2026.2649064 (PMC13081748; doi:10.1080/27694127.2026.2649064)
Supplement: Supplementary_text_2026_final.docx [file KAUO_A_2649064_SM7836.docx]

# Supplementing Information Material and Methods

**DIA-NN parameters**

DIA-NN (version 1.8.1) was run with the following parameters to support dynamic SILAC quantification:

- --fixed-mod SILAC,0.0,KR,label
- --lib-fixed-mod SILAC
- --channels SILAC,L,KR,0:0;SILAC,H,KR,8.014199:10.008269
- --peak-translation
- --original-mods
- --no-norm
- --no-maxlfq
- --peak-center

Additional settings:

- Missed cleavages: 2
- Precursor charge range: 2–4
- Precursor m/z range: 300–1800
- Fragment ion m/z range: 200–1800

# Supplemental figures

**
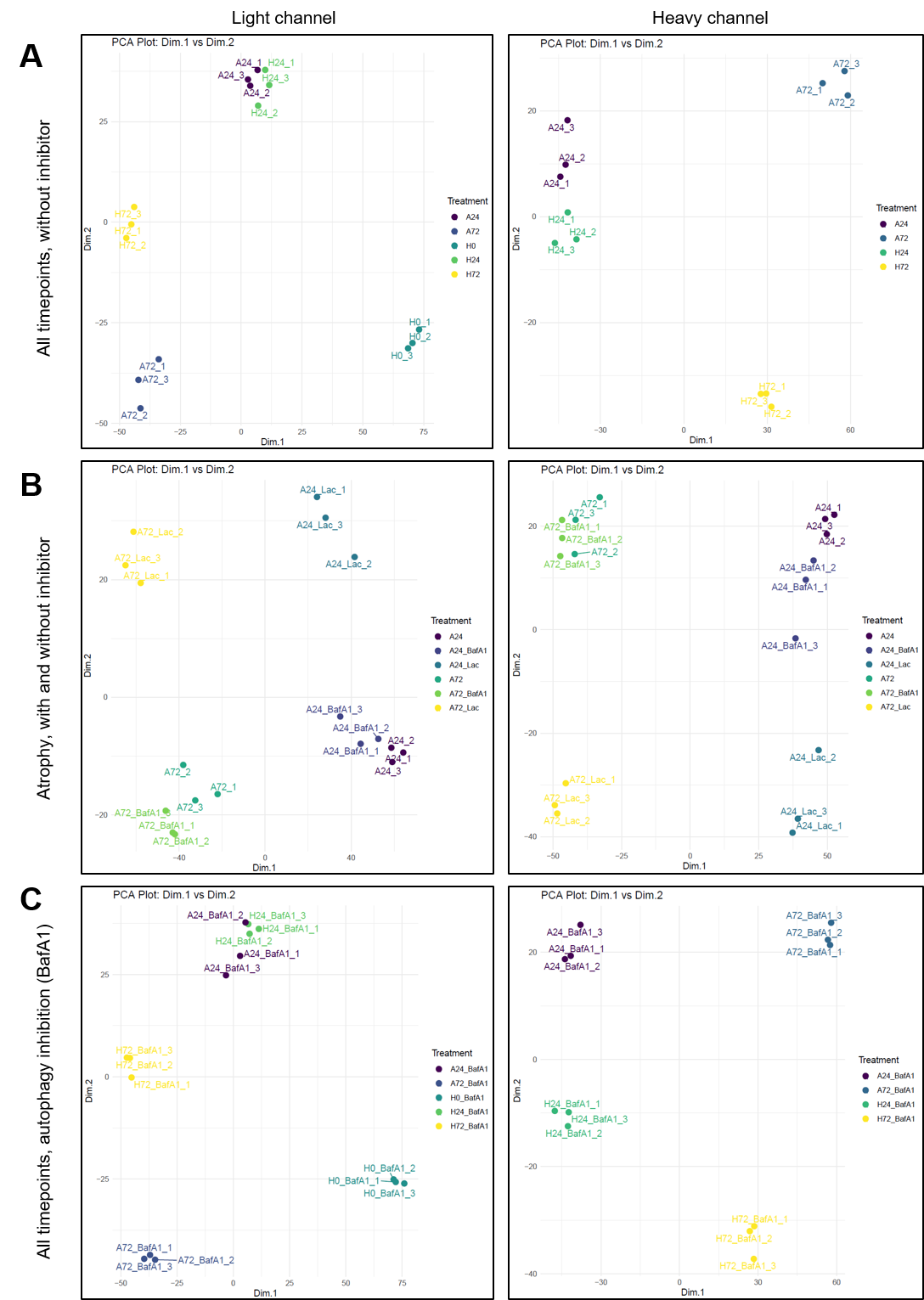
Figure S1**

**Figure S1:** PCA of dynamic SILAC dataset of light and heavy channels comparing (**A**) all time points without proteolytic inhibitors, (**B**) atrophying time points with and without proteolytic inhibitors and (**C)** all time points upon autophagy inhibition. A = atrophy, H = homeostatic conditions, 24 = 24 hours, 72 = 72 hours, 0 = 0 hours with 1-3 depicting three replicates. BafA1 = BafilomycinA1, Lac = Lactacystin.

**Figure S2:**

**
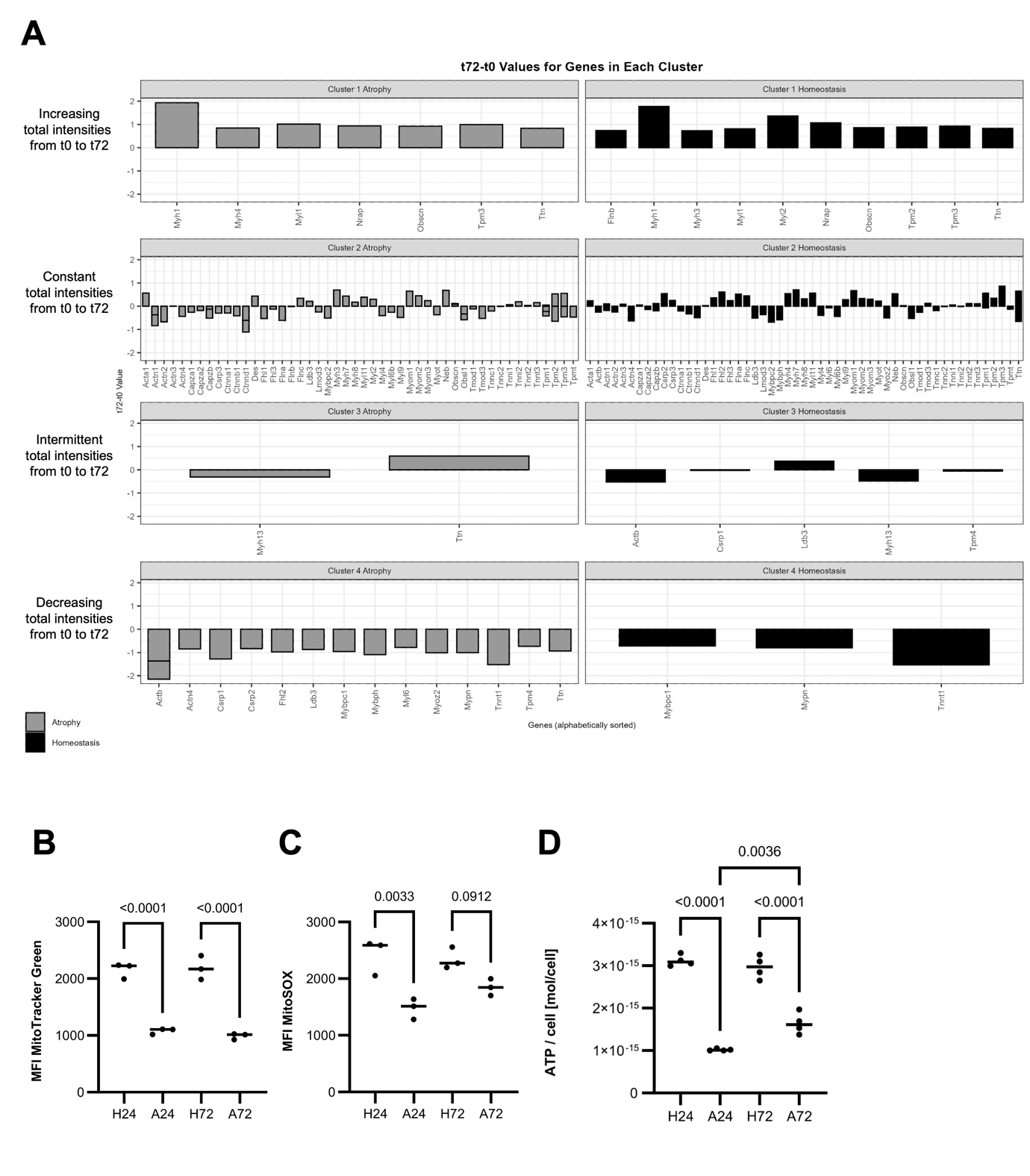
**

**Figure S2:** Temporal profiling of myofibrillar protein expression and autophagy marker quantification. (**A**) Myofibrillar proteins were assigned to clusters based on their temporal profile of total intensities (Heavy + Light) between t0, t24 and t72. Clusters were identified for atrophying (left) and homeostatic (right) conditions. Clusters are defined as: 1: increasing, 2: constant, 3: intermittent, 4: decreasing. Y-axis depicts t72-t0 value. Genes are alphabetically ordered within their clusters. (**B-C**) Flow cytometric analysis of (**A**) mitochondrial mass (MitoTracker Green) and (**B**) mitochondrial superoxide levels (MitoSOX Green). N = 3 replicates. MFI: median fluorescence intensity. (**C**) **Quantification of intracellular ATP levels during TNF-α-induced atrophy:** Cellular ATP content was measured in homeostatic (H24, H72) and atrophying conditions (A24, A72) using a luciferase-based ATP determination kit and normalized to cell number (ATP per cell). N = 4.

**Figure S3:**

**
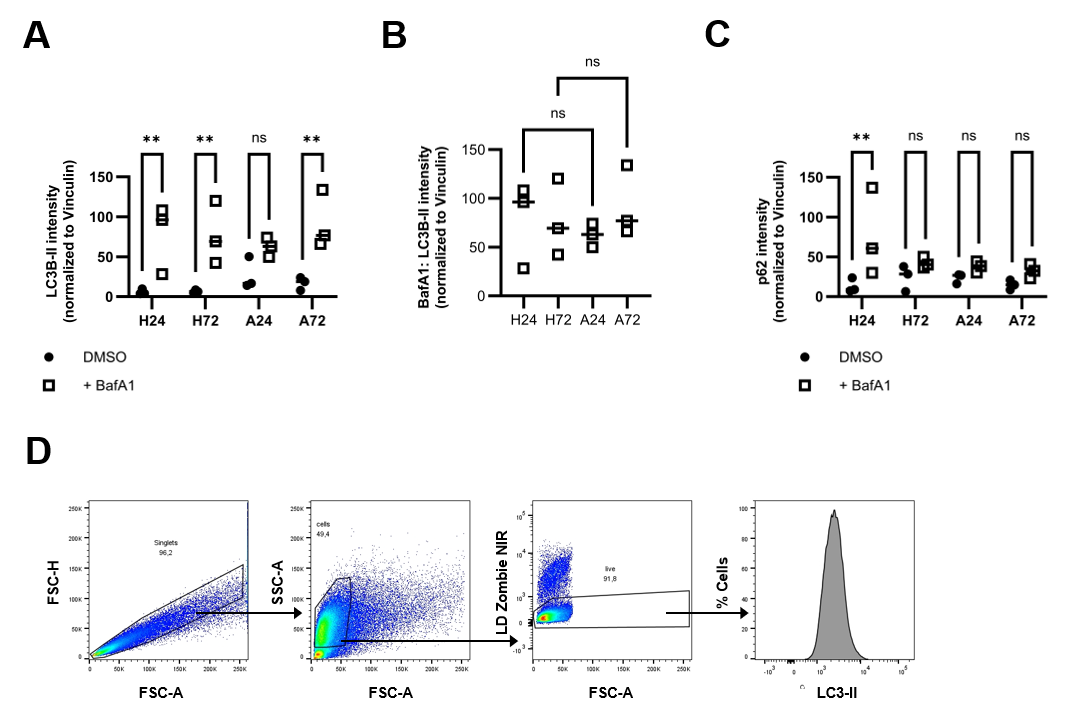
**

**Figure S3:** Quantified Western blot intensities from Figure 5. Timepoints represent homeostatic conditions (H24, H72) and atrophy (A24, A72) at 24 h and 72 h, respectively. ** p < 0.001. ns = not significant. (**A**) LC3B-II intensity normalized to Vinculin in DMSO- and BafA1-treated samples. (**B**) LC3B-II intensity normalized to Vinculin in BafA1-treated samples only. (**C**) p62 intensity normalized to Vinculin in DMSO- and BafA1-treated samples. (**D**) Gating strategy for quantification of LC3-II intensity using flow cytometry. Autophagic turnover was quantified using a flow cytometry-based approach (Alsaleh *et al.*, 2020). For analysis, doublets were excluded first, followed by gating on cells, thus excluding debris and enlarged cells. Next, dead cells were removed by gating on live cells. LC3-II signal was thus only quantified on live, singlet cells.

**Figure S4:**

**
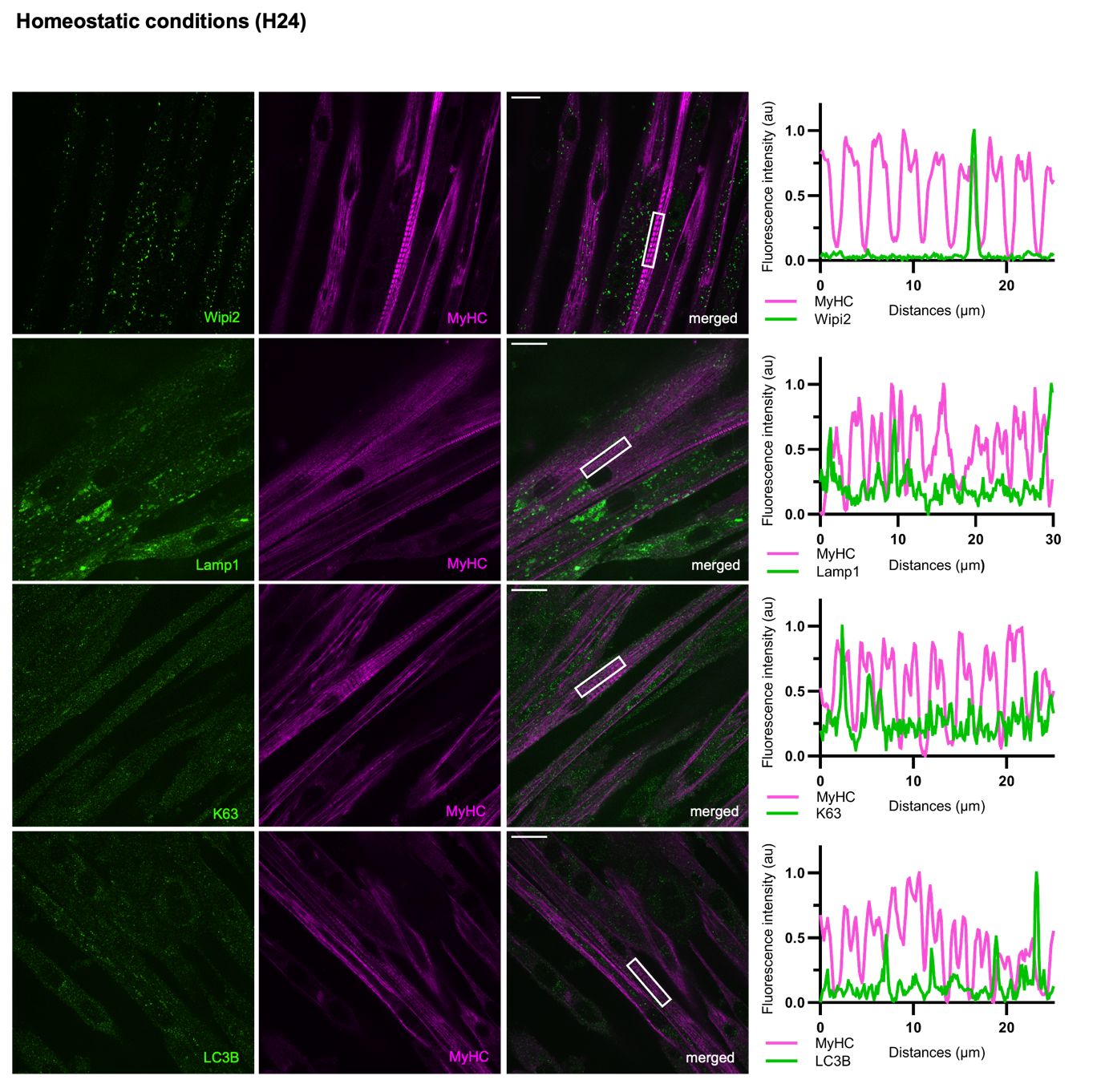
**

**Figure S4:** Autophagy and ubiquitin-related markers do not colocalize with myofibrillar structures under homeostatic conditions. Immunocytochemical stainings for Wipi2, Lamp1, K63-Ub, and LC3B show no colocalization with MyHC (Myosin heavy chain) in homeostatic myotubes with corresponding line plots along the sarcomere. Scale bar: 20 µm.

**Figure S5:
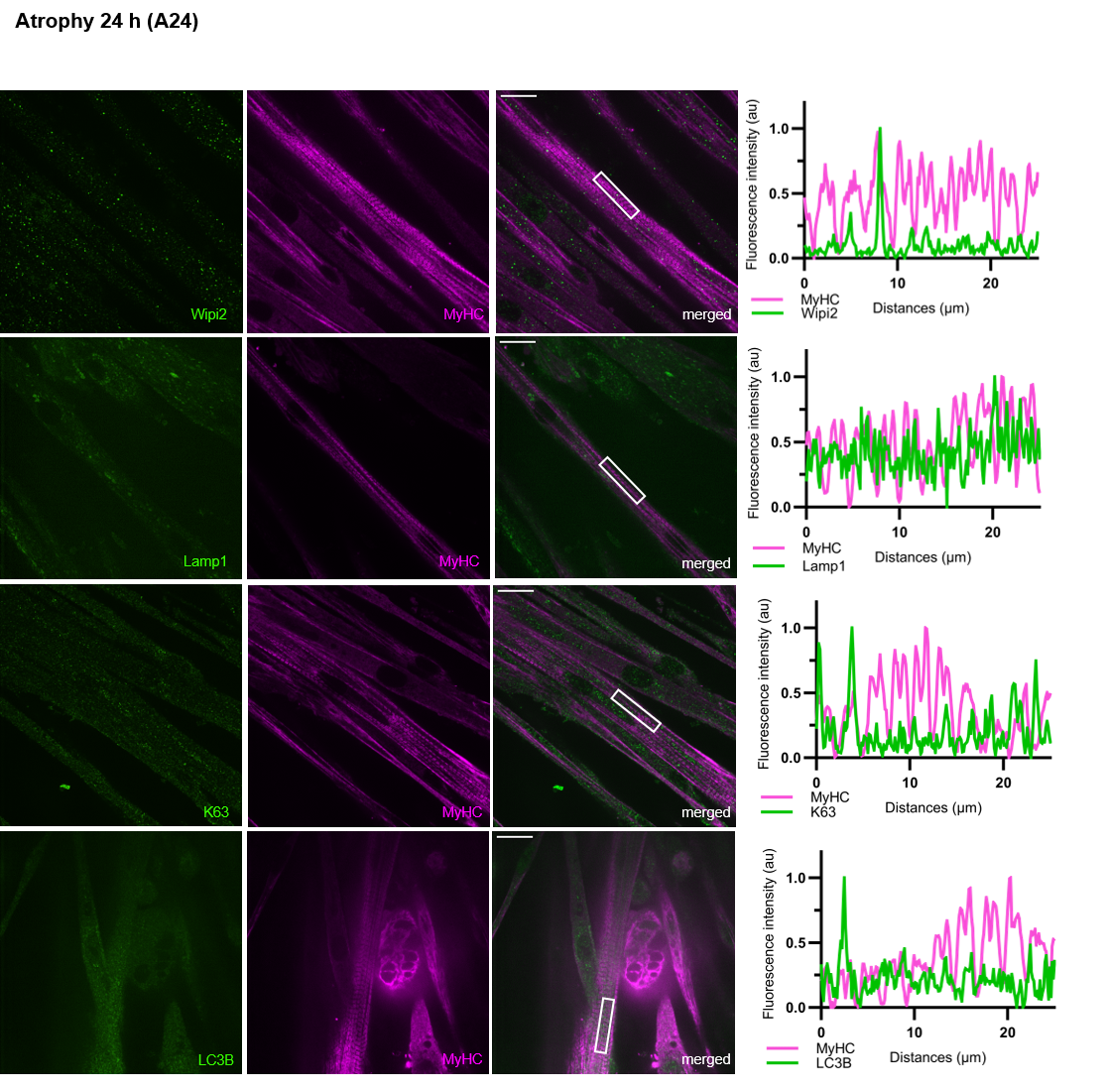
**

**Figure S5:** Autophagy and ubiquitin-related markers do not colocalize with myofibrillar structures under atrophic conditions. Immunocytochemical stainings for Wipi2, Lamp1, K63-Ub, and LC3B show no colocalization with MyHC (Myosin heavy chain) in atrophying myotubes (A24, 24 h TNF-α treatment) with corresponding line plots along the sarcomere. Scale bar: 20 µm.

**Figure S6:**

**
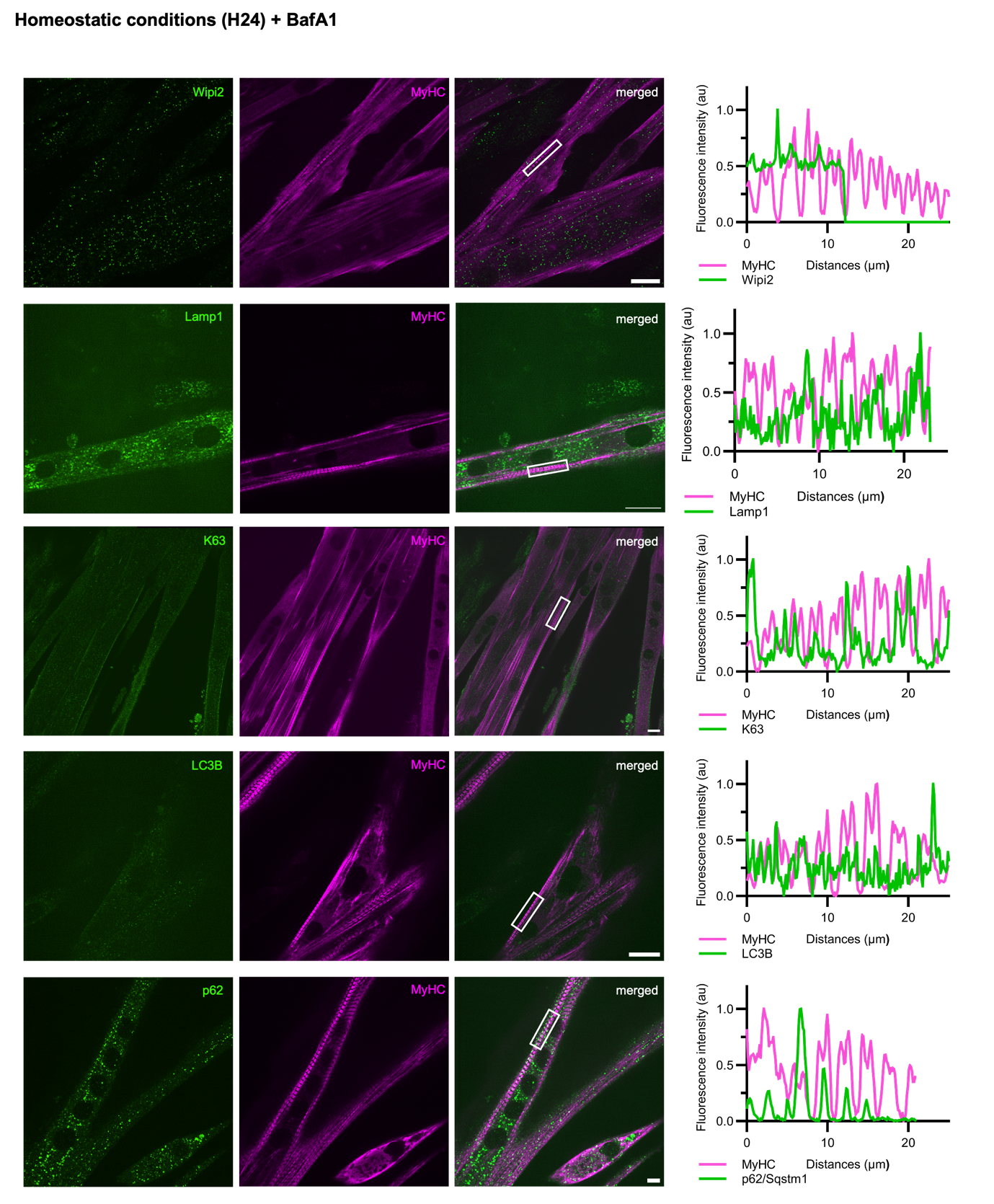
**

**Figure S6:** Only p62, but not other autophagy- or ubiquitin-related markers, specifically localizes with the M band (MyHC, Myosin heavy chain) in homeostatic conditions upon autophagy inhibition (BafA1 treatment): Immunocytochemical stainings for Wipi2, Lamp1, K63-Ub, and LC3B show no specific co-localization with MyHC (Myosin heavy chain) in C2C12 myotubes (H24) as illustrated by corresponding line plots along the sarcomere. Scale bar: 20 µm.

**Figure S7:**

**
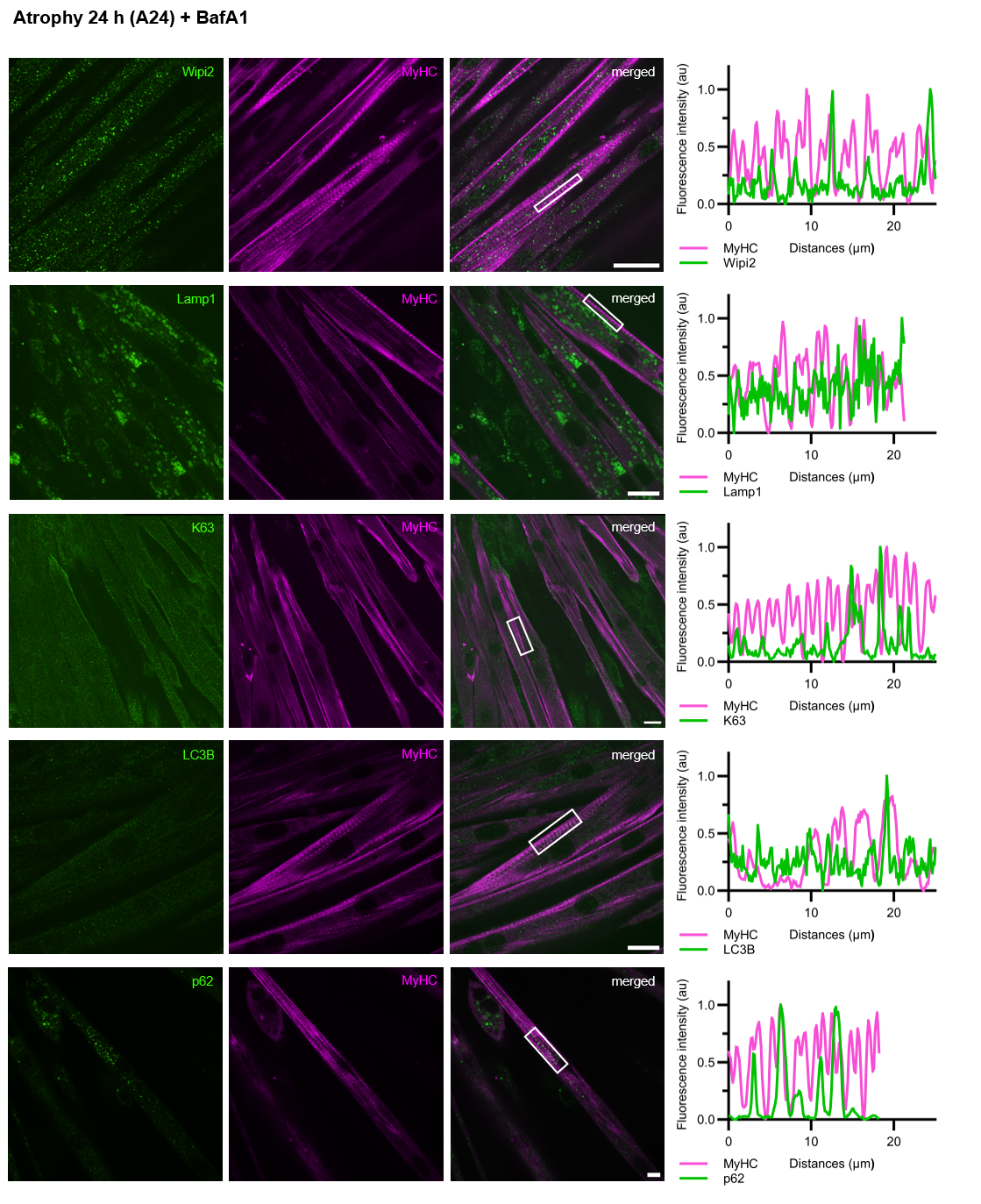
**

**Figure S7:** Only p62, but not other autophagy- or ubiquitin-related markers, specifically localizes with the M band (MyHC, Myosin heavy chain) in atrophy upon autophagy inhibition (BafA1 treatment): Immunocytochemical stainings for Wipi2, Lamp1, K63-Ub, and LC3B show no specific co-localization with MyHC (Myosin heavy chain) in atrophying C2C12 myotubes (A24, 24 h TNF-α treatment) as illustrated by corresponding line plots along the sarcomere. Scale bar: 20 µm.

**Figure S8:**

**
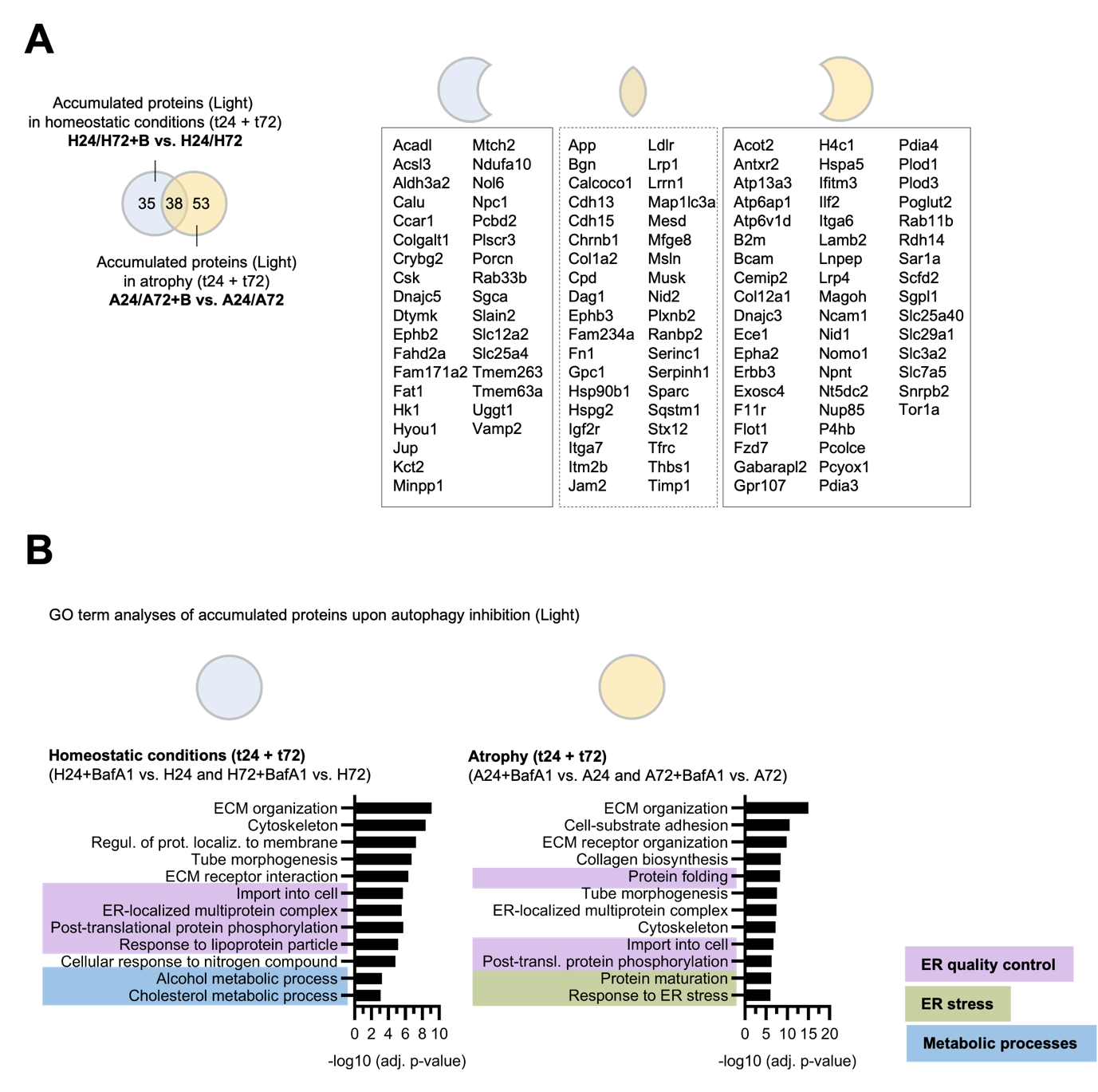
**

**Figure S8:** Proteomic changes upon autophagic inhibition reveal condition-specific accumulation patterns and enriched biological processes. (**A**) ANOVA comparisons of Bafilomycin (BafA1)- vs. non-BafA1-treated samples in homeostasis (both H24 and H72 together) and atrophy (both A24 and A72 together) with log_2_FC > 0 and adj. p-value set at < 0.05. Numbers in Venn diagrams depict amount of significantly regulated proteins in respective dataset comparison. GO term analyses (biological processes) in homeostasis (left) and atrophy (right). Lists depict accumulated proteins upon autophagic inhibition (BafA1) in only homeostasis (H24 + H72) (left), only atrophy (A24 + A72) (right) and proteins accumulating in both conditions (overlap, middle). (**B**) GO term analyses (biological processes) of accumulated proteins upon autophagic inhibition (BafA1) in homeostasis (H24 + H72) and atrophy (A24 + A72).

**Figure S9:**

**
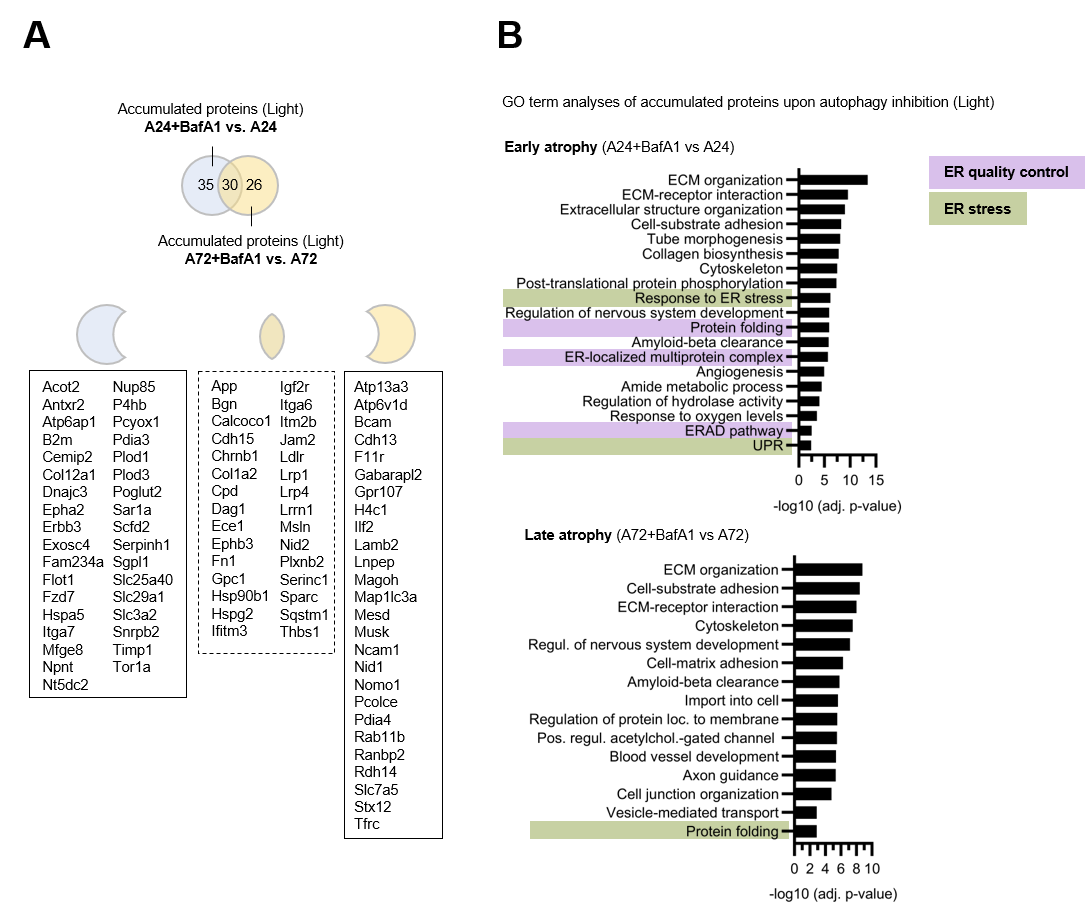
**

**Figure S9:** Stage-specific proteomic responses to autophagic inhibition during early and late muscle atrophy. (**A**) ANOVA comparisons of Bafilomycin (BafA1)- vs. non-BafA1-treated samples in early atrophy (A24+BafA1 vs. A24) and late atrophy (A72+Baf A1 vs. A72) with log_2_FC > 0 and adj. p-value set at < 0.05. Numbers in Venn diagrams depict amount of significantly regulated proteins in respective dataset comparison. Lists depict accumulated proteins in only early atrophy (A24) (left), only late atrophy (A72) (right) and proteins accumulating in both conditions (overlap, middle). (**B**) GO term analyses (biological processes) of accumulated proteins upon autophagic inhibition (BafA1) in early atrophy (top, A24) and late atrophy (bottom, A72).

**Figure S10:**

**
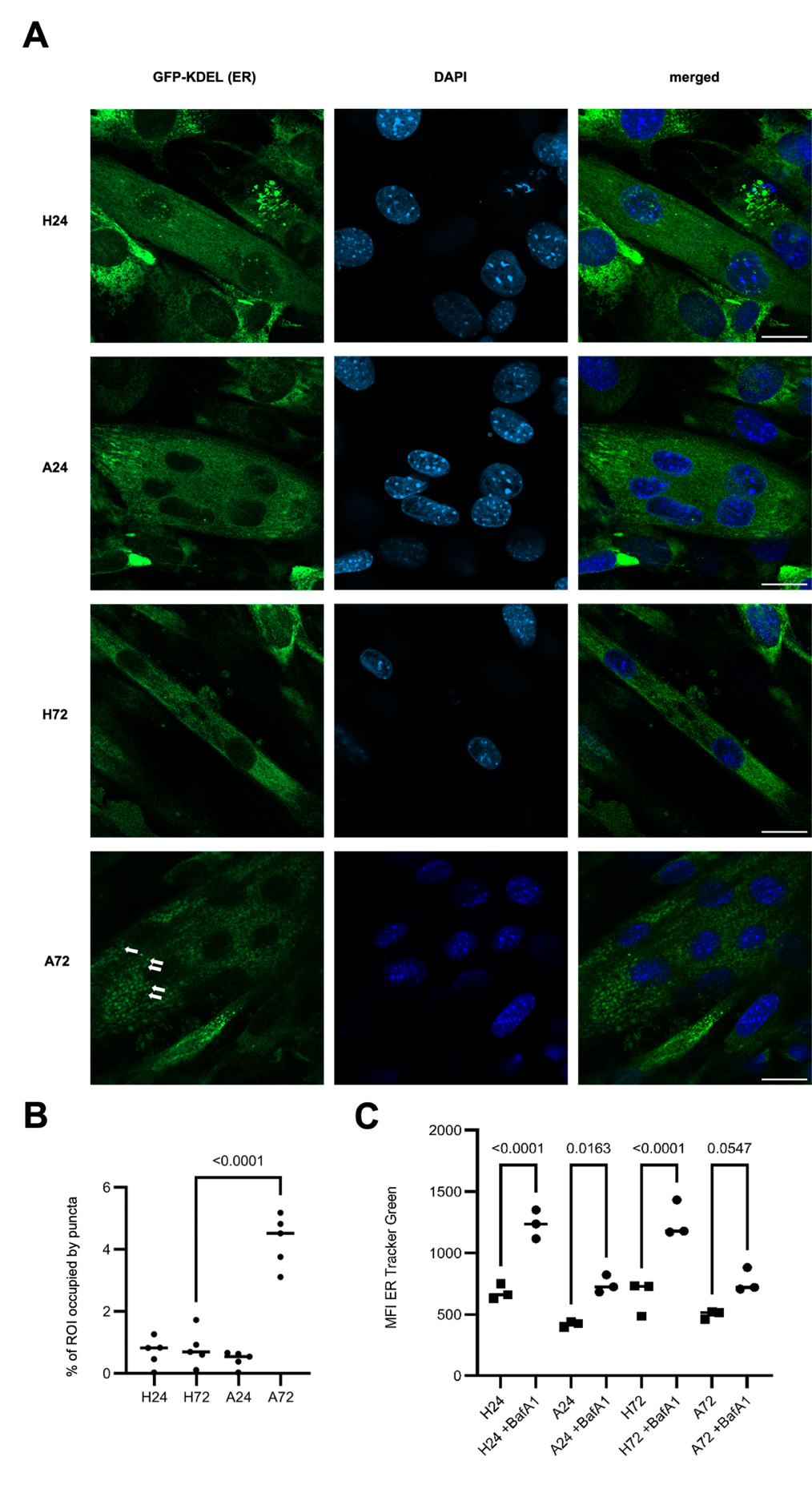
**

**Figure S10: (A)** Visualization of ER morphology with GFP-KDEL-expressing C2C12 myotubes (Buonomo *et al*., 2025) in homeostatic state (H24, H72) and 24 hours and 72 hours of atrophy (A24, A72). KDEL: ER retention signal. Nuclei were stained with DAPI. Scale bar: 20 µm. (**B**) Quantification of (A) showing the percentage of area occupied by GFP-KDEL-positive puncta normalized to the ROI of each cell under under homeostatic (H24, H72) and atrophic (A24, A72) conditions. Puncta were identified using a fixed intensity threshold (20–255) and counted as particles with an area of 1-9 µm². Each dot represents one cell (n = 5 cells per condition); bars indicate mean ± SEM. (**C**) ER abundance in homeostatic conditions (H24, H72) and atrophy (A24, A72) measured with ER-Tracker Green (BODIPY-glibenclamide) via flow cytometry. BafA1: Bafilomycin A1, MFI: median fluorescence intensity. N = 3 replicates.
